# Identification and characterization of circRNAs in peri-implantation endometrium between Yorkshire and Erhualian pigs

**DOI:** 10.1186/s12864-023-09414-1

**Published:** 2023-07-24

**Authors:** Chen Zhou, Xinyan Cheng, Fanming Meng, Yongzhong Wang, Wanyun Luo, Enqin Zheng, Gengyuan Cai, Zhenfang Wu, Zicong Li, Linjun Hong

**Affiliations:** 1 National Engineering Research Center for Breeding Swine Industry, College of Animal Science, South China Agricultural University, Guangzhou, 510642 China; 2 Guangdong Provincial Key Laboratory of Agro-Animal Genomics and Molecular Breeding, South China Agricultural University, Guangzhou, 510642 China; 3 Guangdong Key Laboratory of Animal Breeding and Nutrition, Institute of Animal Science, Guangdong Academy of Agricultural Sciences, Guangzhou, 510640 China; 4 State Key Laboratory of Swine and Poultry Breeding Industry, Guangzhou, 510640 China; 5 Subcenter of Guangdong Laboratory for Lingnan Modern Agriculture, Yunfu, 527300 China

**Keywords:** CircRNA, Erhualian, Yorkshire, Peri-Implantation, Endometrium, Embryo implantation

## Abstract

**Background:**

One of the most critical periods for the loss of pig embryos is the 12th day of gestation when implantation begins. Recent studies have shown that non-coding RNAs (ncRNAs) play important regulatory roles during pregnancy. Circular RNAs (circRNAs) are a kind of ubiquitously expressed ncRNAs that can directly regulate the binding proteins or regulate the expression of target genes by adsorbing micro RNAs (miRNA).

**Results:**

We used the Illumina Novaseq6,000 technology to analyze the circRNA expression profile in the endometrium of three Erhualian (EH12) and three Yorkshire (YK12) pigs on day 12 of gestation. Overall, a total of 22,108 circRNAs were identified. Of these, 4051 circRNAs were specific to EH12 and 5889 circRNAs were specific to YK12, indicating a high level of breed specificity. Further analysis showed that there were 641 significant differentially expressed circRNAs (SDEcircRNAs) in EH12 compared with YK12 (FDR < 0.05). Functional enrichment of differential circRNA host genes revealed many pathways and genes associated with reproduction and regulation of embryo development. Network analysis of circRNA-miRNA interactions further supported the idea that circRNAs act as sponges for miRNAs to regulate gene expression. The prediction of differential circRNA binding proteins further explored the potential regulatory pathways of circRNAs. Analysis of SDEcircRNAs suggested a possible reason for the difference in embryo survival between the two breeds at the peri-implantation stage.

**Conclusions:**

Together, these data suggest that circRNAs are abundantly expressed in the endometrium during the peri-implantation period in pigs and are important regulators of related genes. The results of this study will help to further understand the differences in molecular pathways between the two breeds during the critical implantation period of pregnancy, and will help to provide insight into the molecular mechanisms that contribute to the establishment of pregnancy and embryo loss in pigs.

**Supplementary Information:**

The online version contains supplementary material available at 10.1186/s12864-023-09414-1.

## Introduction

Sow fecundity is an important factor affecting the economic benefits of pig farming, of which litter size is the most important indicator of sow fecundity, which is affected by ovulation number, fertilization rate, embryo mortality, uterine volume and other factors [1, 2]. Although sow fertility is directly related to ovulation rate, the major barrier to increasing litter size of piglets is prenatal mortality [3].

Embryo implantation is a complex process of interaction between the embryo and the endometrium, which involves a variety of kinins, cytokinins and adhesion factors [4]. The porcine morula is formed about 4–5 days after fertilization, and the blastocyst is formed about 6–7 days after fertilization. The embryo migrates from the 8th day to the 12th day of pregnancy and the uterus is equidistant, with dramatic morphological changes from spherical to tubular to filamentous [5]. Porcine embryo implantation begins on the 10th to 13th day of pregnancy, and the conceptuses begin producing estradiol-17 (E_2_) on Days 11–12 of gestation [6], when the porcine endometrium opens the “window period” of implantation, the endometrium is in the receptive state, allowing the blastocyst to attach to it [7], and the embryo secrets signal peptides (such as estradiol 17β) to make the endometrium accept and the embryo implantation begins [8]. The process of embryo implantation is accompanied by a transition in the polarity of the uterine luminal epithelium from a hyperpolar state to a hypopolar state and the secretion of glandular epithelium (GE) [9–11]. The normal endometrium allows embryo implantation only during a very short critical period, which represents the most receptive period of the endometrium and is known as the “implantation window“ [12]. Pig embryos complete implantation around day 18 to day 24 [13, 14]. During the entire gestation of pigs, about 20–45% of embryos are lost during pig gestation, and of these, embryo loss occurs mainly during maternal recognition and implantation [15, 16], with approximately 20–30% of embryo deaths occurring during the peri-implantation period at gestation day (GD) 11–12 [17], which represents a major challenge for the commercial swine industry.

Erhualian (EH) and Meishan (MS), both native Chinese pig breeds belonging to the Taihu Lake (TL) region of eastern China, are well documented for their extraordinary prolificacy [18, 19], among which the reproductive mechanism of Meishan has been studied for more than 30 years, and each litter size is 3 to 5 more than European and North American commercial breeds such as Large White (LW), although they have similar ovulation rates [6]. At a certain level of ovulation rate (OR), the MS breed has more litters by increasing pre-partum survival (PS) [20], and in addition, the Taihu pigs show a high degree of maternal heterogeneity in litter size when crossed with western breeds [21]. Before GD11, there was no difference in embryo survival between different pig breeds, but Meishan pigs had improved embryo survival at GD12 compared to Landrace × Large Yorkshire (LL) [22, 23]. The study found that the higher embryo survival rate of Chinese Taihu pigs at 11–12 days of gestation was the most important factor in promoting the increase in litter size, which was mainly controlled by maternal genes [20, 21, 24]. Many studies have shown that molecular events occurring in the maternal endometrium during early gestation play a crucial role in determining embryo survival success, and the early endometrium provides an ideal window to investigate molecular mechanisms that may be associated with early gestational conception loss and sow reproductive capacity. Especially involved in angiogenesis and expression of some immune factors at the maternal-fetal interface [25, 26]. Interestingly, some studies found that EphA4 [27], TGF-β1 [28] and VEGFA [29] are an important cytokine during embryo attachment and it is also significantly associated with litter size in pigs. Embryo implantation in sows is a complex process of molecular communication between the implanted blastocyst and the recipient uterus, and the molecular mechanisms underlying the differences in embryo implantation rates and development between the two breeds deserve further study.

Circular RNA (circRNA) is a novel class of endogenous non-coding RNA that widely exist in eukaryotes various eukaryotic organisms. Unlike linear RNAs, circRNAs have no 3’-end poly(A) and 5’-end cap structures, and their structures are covalently bonded closed loops at the head and tail [30]. Therefore, it has higher stability and conservativeness than linear RNA, and shows spatio-temporal specificity and tissue specificity in physiological or pathological states [31, 32]. CircRNAs have multiple biological functions, regulating gene expression [33]. Firstly, circRNA is an important competitive endogenous RNA (ceRNA), which has the function of sponge absorbing micro RNAs (miRNAs), that is, it can regulate the expression of genes by competitively binding miRNAs [34]. In addition, circRNAs can also be translated into proteins that regulate gene expression [35]. In recent years, a large number of circRNAs have been successfully identified in a variety of cell lines and species [36], including human endometrium [37], mouse endometrium [38], and goat endometrium [39]. And an increasing number of studies have found that circRNAs play an important role in the regulation of gene expression, the development of disease, and are therefore closely associated with physiological processes and various diseases [40]. In addition, studies have shown that circRNAs are expressed in normal endometrium, endometriosis, and endometrial cancer [41]. Taken together, these results suggest that circRNAs play an important biological regulatory role in the endometrium. In this study, we compared the expression profiles of circRNAs in the endometrium of Erhualian (EH12) and Yorkshire (YK12) pigs at 12 days of gestation using RNase R + RNA-seq technology. Differentially expressed cricRNAs (DEcircRNAs) were identified through bioinformatics analysis and experiments, and the results showed that circRNAs may regulate the key regulators of embryo implantation. Overall, our study contributes to a better understanding of the role of circRNAs in embryo survival during implantation.

## Materials and methods

### Animal and endometrial tissue collection

The experimental animals of this study were Yorkshire and Erhualian pigs, which were raised in the same environment with the same feeding management. Three healthy Yorkshire sows (parity 2) with similar age, genetic background and estrus time and three healthy Erhualian sows (parity 2) with similar age, genetic background and estrus time were selected. All sows were artificially inseminated with fresh semen 12 h after estrus (day 0) and again 24 h later. Endometrial tissue samples were collected from slaughtered sows on day 12 of gestation. Endometrial tissues collected for RNA extraction were rapidly put into liquid nitrogen for further processing and applied for histomorphological observation in paraformaldehyde fixative. Pregnancy was confirmed by the presence of signs of filamentous embryos in the uterine cavity fluid flush of all pregnant sows.

### Hematoxylin-eosin staining (H&E)

Porcine uterine tissue (2-3 cm) was fixed with 4% formaldehyde for one week at room temperature. The fixed tissue was trimmed and then de-watered and waxed. The waxed impregnation tissue was embedded in an embedding machine, and the embedded cooled wax blocks were cut into 4 μm slices on a paraffin microtome. The slices were smoothed out on warm water at 40℃, and then the slides were picked up and baked in an oven at 60℃. Paraffin sections were dewaxed to water in xylene and ethanol with a decreasing concentration gradient, then stained in hematoxylin and eosin, then dehydrated in ethanol with an increasing concentration gradient and xylene, and finally sealed with neutral gum before scanning and imaging under a section scanner to observe morphology and size.

### RNA extraction, library construction, and circRNA sequencing

Total RNAs was extracted using Trizol reagent kit (Invitrogen, Carlsbad, CA, USA) according to the manufacturer’s protocol. RNA quality was assessed on an Agilent 2100 Bioanalyzer (Agilent Technologies, Palo Alto, CA, USA) and checked using RNase free agarose gel electrophoresis. After extracted, total RNAs were treated with RNase R to degrade the linear RNAs, and purified using RNeasy MinElute Cleanup Kit (Qiagen,Venlo, The Netherlands). Next, strand-specific library was constructed using VAHTS Total RNAseq (H/M/R) Library Prep Kit (Vazyme, Nanjing, China) for Illumina following the manufacture’s instructions. Briefly, ribosome RNAs were removed to retain circRNAs. The enriched circRNAs were fragmented into short fragments by using fragmentation buffer and reverse transcribed into cDNA with random primers. Second-strand cDNA were synthesized by DNA polymerase I, RNase H, dNTP (dUTP instead of dTTP) and buffer. Next, the cDNA fragments were purified with VAHTSTM DNA Clean Beads (Vazyme, Nanjing, China), end repaired, A base added, and ligated to Illumina sequencing adapters. Then UNG (Uracil-N-Glycosylase) was used to digest the second-strand cDNA. The digested products were purified with VAHTSTM DNA Clean Beads, PCR amplified, and sequenced using Illumina Novaseq6,000 by Gene Denovo Biotechnology Co. (Guangzhou, China).

### Pre-processing of sequencing reads and quality control

Reads obtained from the sequencing machines included raw reads containing adapters or low quality bases which would affect the following analysis. Thus, to get high quality clean reads, reads were further filtered by fastp (version 0.18.0) [42]. The parameters were as follows: (1) removing reads containing adapters; (2) removing reads containing more than 10% of unknown nucleotides (N); (3) removing low quality reads containing more than 50% of low quality (*q*-value ≤ 20) bases.

Different species and sample qualities would affect the efficiency of experimental ribosome RNA removal. Thus, short reads alignment tool Bowtie2(version 2.2.8) was used for mapping reads to ribosome RNA (rRNA) database [43]. The rRNA mapped reads will be removed. The remaining reads were further used in alignment and analysis.

The rRNA removed reads of each sample were then mapped to reference genome by TopHat2(version 2. 1.1) [44], respectively. After aligned with reference genome, the reads that could be mapped to the genomes were discarded, and the unmapped reads were then collected for circRNA identification.

### Identification of circRNA

20mers from both ends of the unmapped reads were extracted and aligned to the reference genome to find unique anchor positions within splice site. Anchor reads that aligned in the reversed orientation (head-to tail) indicated circRNA splicing and then were subjected to find_circ (version 1) to identify circRNAs [45]. The anchor alignments were then extended such that the complete read aligns and the breakpoints were flanked by GU/AG splice sites. A candidate circRNA was called if it was supported by at least two unique back spliced reads at least in one sample.

### Quantification of circRNA abundance

To quantify circRNAs, back-spliced junction reads were scaled to RPM (Reads Per Million mapped reads) [46], and the formula is shown as follows:

$$RPM=\frac{{10}^{6}C}{N}$$

In this formula, C is the number of back-spliced junction reads that uniquely aligned to a circRNA. N is the total number of back-spliced junction reads. The RPM method is able to eliminate the influence of different sequencing data amount on the calculation of circRNA expression. Therefore, the calculated expression can be directly used for comparing the differential expression among samples.

### Analysis of differentially expressed circRNAs

To identify DEcircRNAs between EH12 and YK12 pigs, the edgeR package (version 3.12.1) (http://www.r-project.org/) was used. We identified circRNAs with a fold change ≥ 2 and a FDR < 0.05 in a comparison between EH12 and YK12 as significant differentially expressed circRNAs (SDEcircRNAs).

#### GO enrichment analysis

Gene Ontology (GO) is an international standardized gene functional classification system which offers a dynamic-updated controlled vocabulary and a strictly defined concept to comprehensively describe properties of genes and their products in any organism [47]. GO has three ontologies: molecular function, cellular component and biological process. The basic unit of GO is GO-term. Each GO-term belongs to a type of ontology. GO enrichment analysis provides all GO terms that significantly enriched in host genes comparing to the genome background, and filter the host genes that correspond to biological functions. Firstly, all host genes were mapped to GO terms in the Gene Ontology database (http://www.geneontology.org/), gene numbers were calculated for every term, significantly enriched GO terms in host genes comparing to the genome background were defined by hypergeometric test. The calculating formula of *P*-value is as follows:

$$P=1-\sum _{i=0}^{m-1}\frac{\left(\begin{array}{c}M\\ i\end{array}\right)\left(\begin{array}{c}N-M\\ n-i\end{array}\right)}{\left(\begin{array}{c}N\\ n\end{array}\right)}$$

Here N is the number of all genes with GO annotation; n is the number of host genes in N; M is the number of all genes that are annotated to the certain GO terms; m is the number of host genes in M. The calculated *P*-value were gone through FDR (False Discovery Rate) Correction, taking FDR ≤ 0.05 as a threshold. GO terms meeting this condition were defined as significantly enriched GO terms in host genes. This analysis was able to recognize the main biological functions that host genes exercise.

### Pathway enrichment analysis

Genes usually interact with each other to play roles in certain biological functions. Pathway-based analysis helps to further understand genes biological functions. KEGG (Kyoto Encyclopedia of Genes and Genomes) is the major public pathway-related database [48]. Pathway enrichment analysis identified significantly enriched metabolic pathways or signal transduction pathways in host genes comparing with the whole genome background. The calculating formula is the same as that in GO analysis:

$$P=1-\sum _{i=0}^{m-1}\frac{\left(\begin{array}{c}M\\ i\end{array}\right)\left(\begin{array}{c}N-M\\ n-i\end{array}\right)}{\left(\begin{array}{c}N\\ n\end{array}\right)}$$

Here N is the number of all genes that with KEGG annotation, n is the number of host genes in N, M is the number of all genes annotated to specific pathways, and m is number of host genes in M. The calculated *P*-value was gone through FDR Correction, taking FDR ≤ 0.05 as a threshold. Pathways meeting this condition were defined as significantly enriched pathways in host genes.

### Motif enrichment analysis

The meme-chip (version 5.0.5) MEME tool was used to retrieve long motifs of 8-15 bp from the input sequence, and the Dreme tool was used to retrieve short motifs of 3-8 bp from the JASPAR database. The retrieved motifs were then compared with known motifs in the JASPAR database using meme-chip Tomtom to filter out known motifs that were similar to the retrieved motifs. Finally, the position of the motif in the input sequence is located and visualized using meme-chip fimo.

### miRNA prediction and construction of the ceRNA network

For circRNAs that have been annotated in circBase, the target relationship with miRNAs can be predicted by StarBase (version 2.0). For novel circRNAs, three softwares Mireap, Miranda (version 3.3a) and TargetScan (version 7.0) were used to predict targets for our samples, And the resulting correlation of circRNAs-miRNAs can be visualized by Cytoscape.

### Reverse transcription PCR (RT-PCR) analysis and sanger sequencing

Total endometrial RNA was extracted using the Trizol method and endometrial tissue DNA was extracted using the OMEGA Tissue DNA Kit (D3396). The extracted total RNA was used to synthesize cDNA according to the instructions of the TAKARA Reverse Transcription Kit PrimeScript® RT reagent Kit With gDNA Eraser (RR047A). The cDNA and tissue DNA were subjected to PCR using convergent and divergent primers, and the PCR products were subjected to gel electrophoresis and Sanger sequencing to verify the junction sequence of the circRNA.

### Analysis of the resistance of circRNAs to RNase R treatment

We divided the extracted endometrial total RNA samples into RNaseR enzyme treatment group and control group, and cDNA was synthesized according to the instructions of TAKARA reverse transcription kit PrimeScript® RT reagent Kit With gDNA Eraser (RR047A). Then, the expression of circRNA between these two groups was detected by real-time quantitative PCR to test the resistance of circRNA to RNaseR enzyme. The fluorescence quantification of circRNA adopts special divergent primers, and the *GAPDH* gene is used as an internal control. The method of fluorescence quantification was according to the instructions of PowerUp SYBR Green Master Mix Kit(A25741), Fluorescence quantitative data was analyzed using Excel software, relative gene expression was analyzed using 2^−ΔΔCt^ value, and the significant difference between groups was analyzed using independent sample t test.

### Quantitative real-time PCR (qPCR) analysis

To verify the accuracy of sequencing quantification, we also used real-time quantitative PCR to verify the differential expression of circRNAs between peri-implantation endometrial sample groups. Fluorescence quantification of circRNA was performed using divergent primers, with the GAPDH gene as an internal reference. Fluorescence quantification was performed according to the instructions of PowerUp SYBR Green Master Mix Kit (A25741). Fluorescence quantification data were analyzed by Excel software, and relative gene expression was analyzed by 2^−ΔΔCt^ values, and the significance of differences between groups was determined using independent sample t-tests.

## Results

### Comparison of endometrial morphology and correlation between Yorkshire and Erhualian

**Fig. 1:**
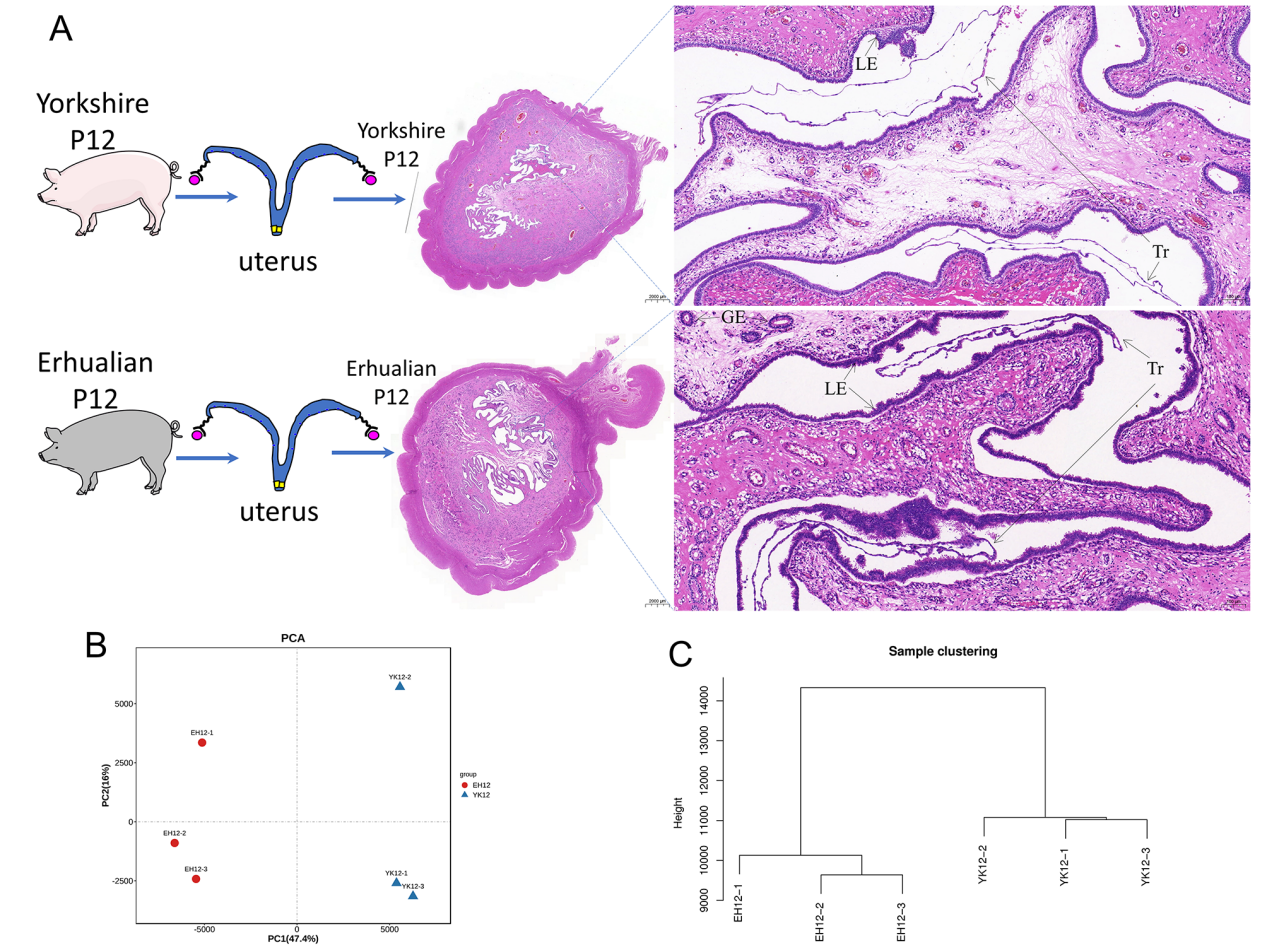
Comparison of Yorkshire and Erhualian uterus samples. (**A**) Comparison of uterine morphology between Yorkshire and Erhualian during peri-implantation. PCA (**B**) and cluster dendrogram (**C**) characterize the clustering distribution of Erhualian and Yorkshire endometrium samples, respectively

We collected uterine tissue slices from Yorkshire pigs and Erhualian pigs during the peri-implantation period and characterized their morphology with hematoxylin and eosin staining (Fig. 1A). Through the visual evaluation of the uterine slices, we found that the embryos of Erhualian pigs and Yorkshire pigs at 12 days of gestation were both in the peri-implantation stage, and the endometrium was folded into the uterine cavity and presented dense ridges. The conceptus have differentiated into filaments and are about to adhere to the endometrial surface. Compared with Yorkshire pigs, the uterus of Erhualian pigs was more flushed, the endometrial stroma had larger blood vessels and more glands, and the uterine cavity epithelial cells showed more and thicker sticky chorion. Additionally, we performed principal component analysis (PCA) on the sequencing raw data. The results of PCA showed that the data of EH12 can be clearly distinguished from the samples of YK12 pigs (Fig. 1B), and the cluster dendrogram will further show that there are obvious differences in the endometrium of Erhualian and Yorkshire pigs at the peri-implantation stage (Fig. 1C).

### Dynamic changes of circRNAs transcripts in Yorkshire and Erhualian endometrium

**Fig. 2:**
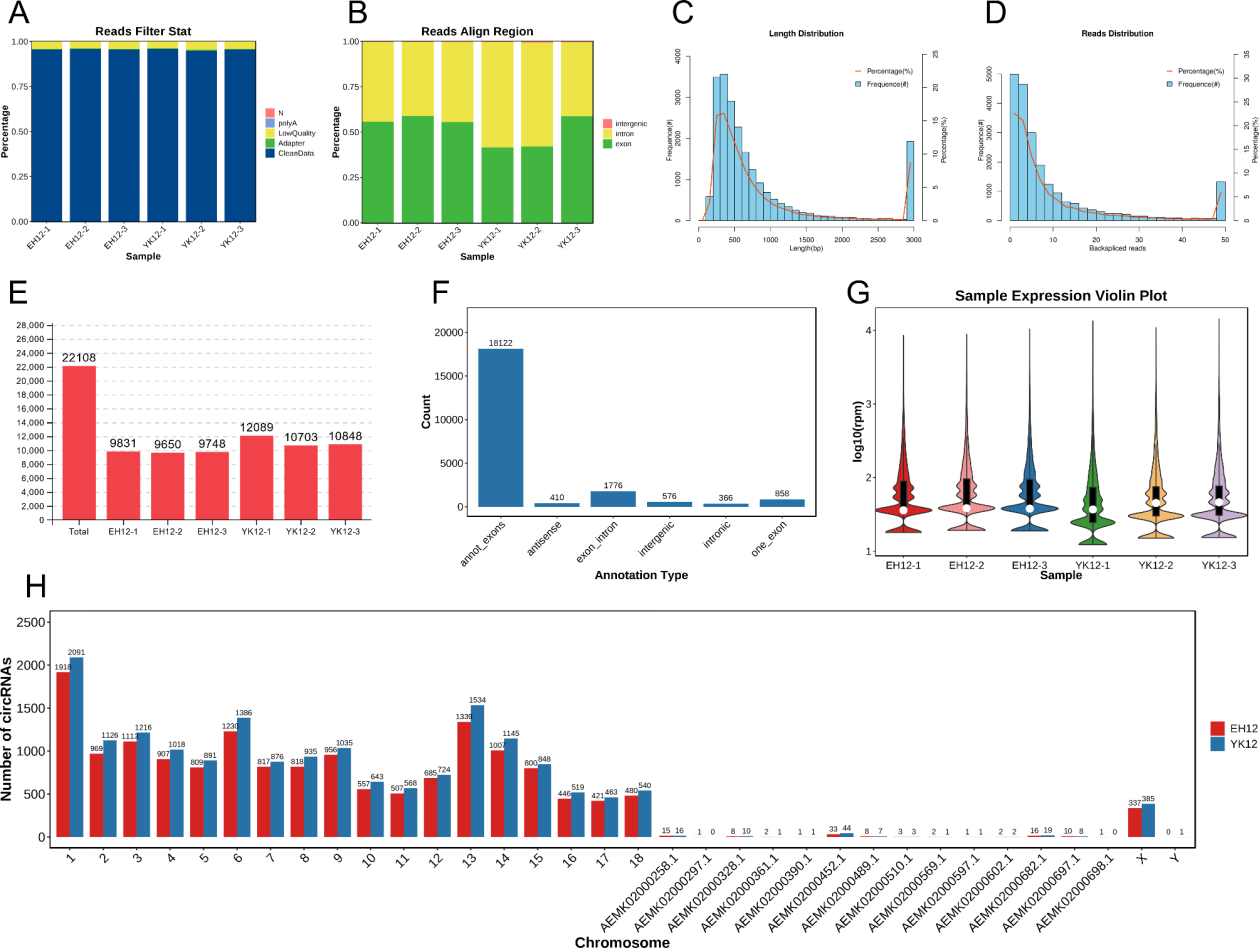
Characterization of circRNA expression in the endometrium of Yorkshire and Erhualian pigs during the peri-implantation period. (**A**) Filter Statistics Percentage. (**B**) spliced reads comparison reference area statistics. (**C**) Length distribution of circRNAs. (**D**) Frequency of back-splicing reads of circRNAs identified in endometrial tissues of 3 Yorkshire and 3 Erhualian pigs. (**E**) The number of circRNAs identified in each sample of Yorkshire and Erhualian endometrium. (**F**) The genomic origin of porcine circRNAs. (**G**) Violin plot of relative abundance of circRNAs in Yorkshire and Erhualian endometrial tissue. Data are expressed as log2 changes in RPM. White dots represent medians. (**H**) Chromosome distribution of the sequences from which circRNAs were derived

To assess the dynamics of circRNA expression in the endometrium of Yorkshire and Erhualian pigs at the peri-implantation stage, we quantified effective reads in endometrial RNA-sequencing data after RNase R treatment. We generated a total of 555 million paired-end reads of 150 bp in length from the endometrium of six sows using the Illumina paired RNA-Seq method (Table 1). This method produced a sequence of 34.8Gb. In order to ensure the quality of the data, we used fastp to perform quality control on the raw reads off the machine, and obtained clean reads. we found that CleanData accounted for about 95% of the raw data (Fig. 2A). Then we calculate the distribution position of reads in the reference genome, and we divide the region compared to the genome into exon region and intron region, each accounting for about 50%, and intergenic region accounted for less than 1% (Fig. 2B). A detailed summary of each sample is provided in Table 1. Analysis of the length distribution of identified circRNAs revealed that most exonic circRNAs were less than 1500 nucleotides (nt) in length, with a median length of approximately 500 nt (Fig. 2C). All identified circRNAs contained at least two unique back-splicing reads (Fig. 2D). Overall, a total of 22,108 unique candidate circRNAs were identified in these tissues (Fig. 2E). Interestingly, we found that more than 80% of circRNAs composed of internal exons, rather than the first and last exons within the same host gene, and a smaller fraction with were circRNAs composed of exon-intron, single exons, intergenic regions in the genome, antisense region sequences of known transcripts and intronic sequences (Fig. 2F). Use violin plots to identify overall circRNA expression outliers and assess overall quality. The violin plot distribution of log 10 RPM values indicated a different expression distribution between YK12 samples and EH12 samples, and our results showed that YK12 was expressed at a slightly lower level than EH12 (Fig. 2G). By further comparing the expression levels of the two groups of samples, we can calculate the difference in circRNA expression. For the distribution of circRNAs on chromosomes, the distribution of circRNAs was found on all 20 chromosomes, and the number of circRNAs on each chromosome was visualized in Fig. 2H.

**Table 1:** The overview of the results of RNA-Seq in each sample

Data filtering statistics			Ribosome alignment statistics			Reference genome alignment statistics			
Sample	RawDatas	CleanData(%)	clean_reads	Mapped_Reads(%)	Unmapped_Reads(%)	Total	Unmapped(%)	Unique_Mapped(%)	Multiple_Mapped(%)
EH12-1	85,530,130	81,622,174 (95.43%)	81,622,174	152,792 (0.19%)	81,469,382 (99.81%)	81,469,382	31,846,412 (39.09%)	29,591,731 (36.32%)	20,031,239 (24.59%)
EH12-2	97,989,130	93,802,044 (95.73%)	93,802,044	559,090 (0.60%)	93,242,954 (99.40%)	93,242,954	36,046,670 (38.66%)	33,607,197 (36.04%)	23,589,087 (25.30%)
EH12-3	89,568,894	85,463,090 (95.42%)	85,463,090	250,724 (0.29%)	85,212,366 (99.71%)	85,212,366	35,349,791 (41.48%)	29,487,562 (34.60%)	20,375,013 (23.91%)
YK12-1	92,203,074	88,358,158 (95.83%)	88,358,158	193,166 (0.22%)	88,164,992 (99.78%)	88,164,992	33,033,217 (37.47%)	32,445,726 (36.80%)	22,686,049 (25.73%)
YK12-2	86,829,768	82,295,580 (94.78%)	82,295,580	173,708 (0.21%)	82,121,872 (99.79%)	82,121,872	35,209,663 (42.87%)	29,264,605 (35.64%)	17,647,604 (21.49%)
YK12-3	102,897,342	98,369,666 (95.60%)	98,369,666	441,796 (0.45%)	97,927,870 (99.55%)	97,927,870	39,430,888 (40.27%)	35,851,130 (36.61%)	22,645,852 (23.13%)

### Comparison of highly expressed circRNAs in the endometrium of Yorkshire and Erhualian

**Fig. 3:**
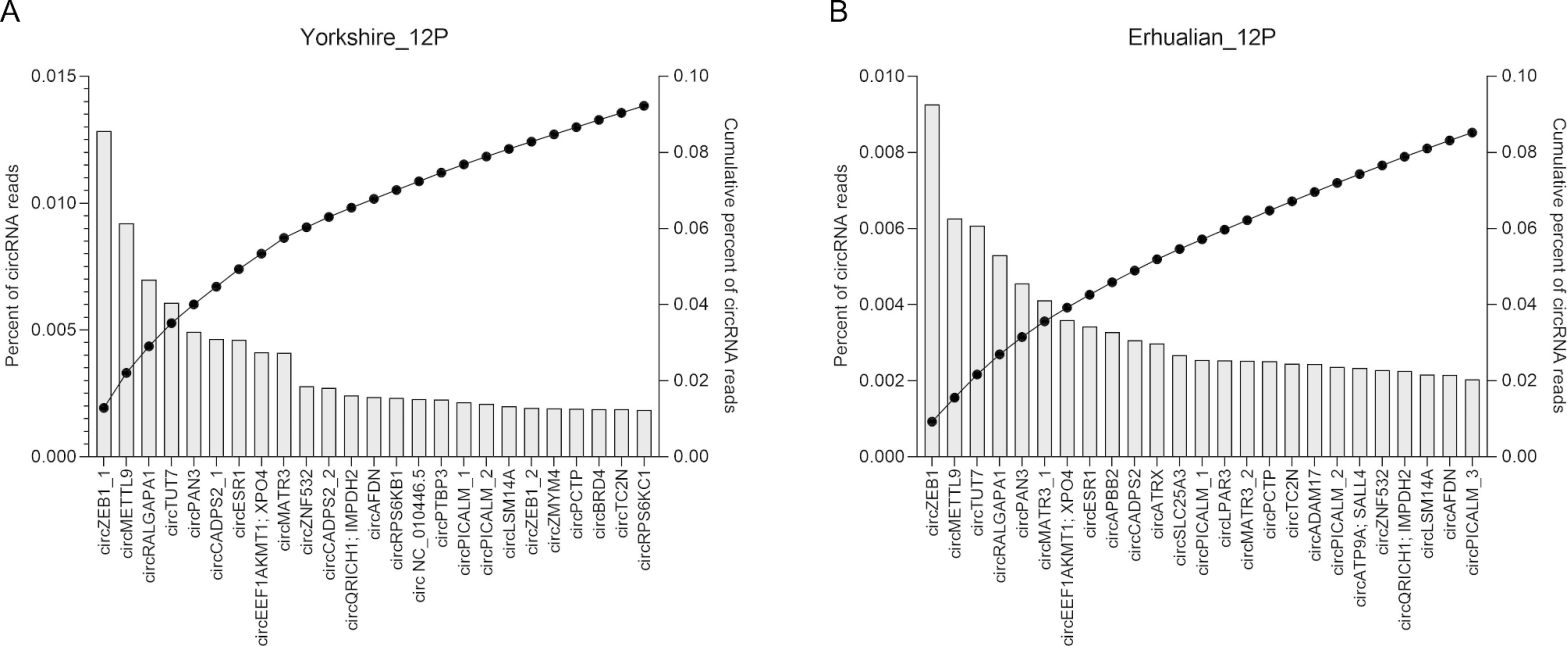
The top 25 circRNAs with the highest content in Yorkshire (**A**) and Erhualian (**B**) endometrium. Left axis and histogram: Percentage of each circRNA to total circRNA reads. Right axis and dots: cumulative percentage of circRNA reads

To characterize the highly abundant circRNAs in YK12 and EH12 endometrium, we ranked all identified circRNAs by abundance. In Yorkshire pigs, circZEB1 was the most abundant, accounting for 12.84% of the total circRNAs, the top five circRNAs (circZEB1, circMETTL9, circRALGAPA1, circTUT7, circPAN3) accounted for 40.04% of the total circRNAs, and the top 25 circRNAs Accounted for 92.25% of the total circRNAs (Fig. 3A). In Erhualian pigs, circZEB1 was the most abundant, accounting for 9.26% of the total circRNAs, the top 5 circRNAs (circZEB1, circMETTL9, circTUT7, circRALGAPA1, circPAN3) accounted for 31.46% of the total circRNAs, and the top 25 circRNAs Accounted for 85.19% of the total circRNAs (Fig. 3B).

### Differential expression of circRNA in Yorkshire and Erhualian endometrium

**Fig. 4:**
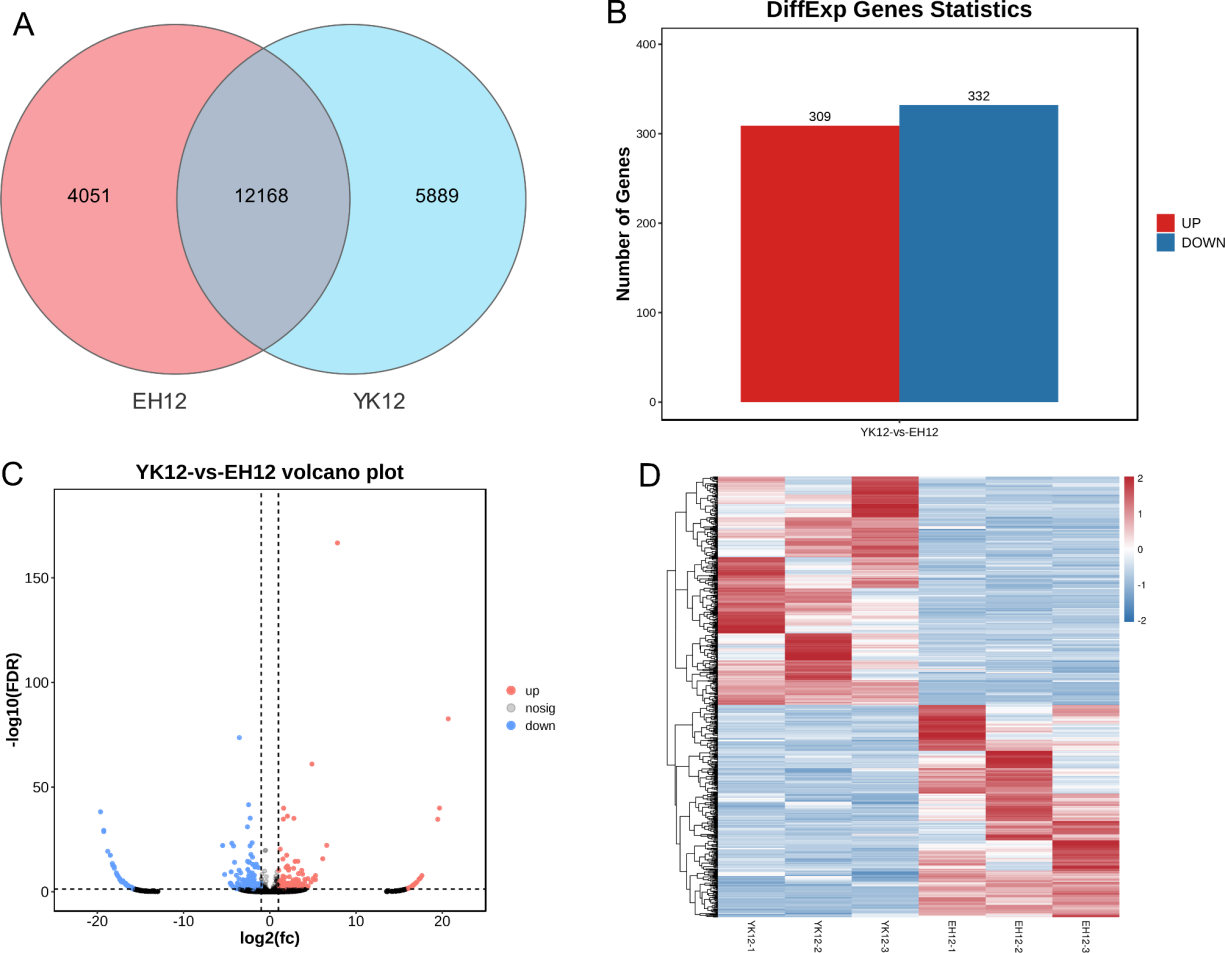
Expression profiles of DEcircRNAs. (**A**) Number of unique circRNAs tags between Yorkshire and Erhualian. (**B**) Histogram of SDEcircRNAs between Yorkshire and Erhualian. (**C**) Volcano map of SDEcircRNAs. (**D**) Clustering heatmap of DEcircRNAs.

To gain insight into the differential expression patterns of endometrial circRNAs in different breeds during the peri-implantation period, we performed a systematic cluster analysis of the data on DEcircRNAs in EH12 and YK12 endometrium. Of all circRNAs identified, we found 5889 circRNAs specifically expressed in YK12, 4051 circRNAs specifically expressed in EH12, and 12,168 circRNAs co-expressed in both groups (Fig. 4A). For EH12 compared with YK12, we found 641 SDEcircRNAs (Supplementary Table S1), of which 309 were up-regulated, and 332 down-regulated (Fig. 4B,C). The heat map clearly demonstrates this differential expression pattern, with samples within the YK12 and EH12 groups clustered separately because their intra-group samples have similar expression profiles; samples between the YK12 and EH12 groups are clustered in another category (Fig. 4D), and the top 25 up-regulated and down-regulated circRNAs were identified based on log_2_FC values (Table 2).

**Table 2:** Top 25 significantly upregulated and downregulated endometrial circRNA in EH12 sows compared to YK12 sows

Top 25 up-regulated					Top 25 down-regulated				
circRNA Symbol	id	log2(fc)	FDR	host_gene	circRNA Symbol	id	log2(fc)	FDR	host_gene
ssc_circ:chr7:20896458_20904929(+)	ssc_circ:chr7:20896458_20904929(+)	20.6864	2.23E-83	ENSSSCG00000049262	circTMEM237	ssc_circ:chr15:105263579_105266463(-)	-19.63463	6.15E-39	TMEM237
circPRDM2_1	ssc_circ:chr6:73137415_73166221(+)	19.6808	9.85E-41	PRDM2	ssc_circ:chr9:136959931_136972049(-)	ssc_circ:chr9:136959931_136972049(-)	-19.25721	1.69E-29	ENSSSCG00000015632
circPRDM2_2	ssc_circ:chr6:73137415_73172671(+)	19.4759	2.28E-35	PRDM2	circEP300	ssc_circ:chr5:7320472_7321718(-)	-19.25587	4.67E-30	EP300
circFHOD3	ssc_circ:chr6:120105191_120120511(+)	17.6395	1.78E-08	FHOD3	ssc_circ:chr8:74002087_74002241(+)	ssc_circ:chr8:74002087_74002241(+)	-18.79684	4.45E-20	ENSSSCG00000049801
circTCF12	ssc_circ:chr1:114880144_114881141(-)	17.4531	2.47E-07	TCF12	circAP2B1	ssc_circ:chr12:39904915_39918403(-)	-18.49514	3.32E-18	AP2B1
circKCTD3_1	ssc_circ:chr10:5761746_5777849(+)	17.412	4.77E-07	KCTD3	circEXOC4	ssc_circ:chr18:15347146_15348948(-)	-18.25553	3.14E-14	EXOC4
circGRM8_1	ssc_circ:chr18:21301169_21303712(+)	17.3018	8.82E-07	GRM8	circADGRA3	ssc_circ:chr8:16728875_16732088(-)	-18.21564	2.21E-13	ADGRA3
circZEB1	ssc_circ:chr10:42185715_42194913(+)	17.0659	1.99E-05	ZEB1	circCWH43_1	ssc_circ:chr8:38873398_38914541(+)	-18.06138	7.96E-13	CWH43
ssc_circ:chr13:173598529_173601872(-)	ssc_circ:chr13:173598529_173601872(-)	16.9175	7.06E-05	NA	ssc_circ:chr4:76518127_76546356(-)	ssc_circ:chr4:76518127_76546356(-)	-18.03153	5.42E-12	NA
circHHLA2	ssc_circ:chr13:151200864_151265264(-)	16.8998	7.06E-05	HHLA2	circKLRK1	ssc_circ:chr5:61618309_61628294(+)	-17.80284	9.47E-10	KLRK1
circGRM8_2	ssc_circ:chr18:20960752_20961555(+)	16.7915	0.000237648	GRM8	circRALA	ssc_circ:chr18:54558710_54563773(-)	-17.71786	6.44E-09	RALA
circSPATA6	ssc_circ:chr6:163299858_163384931(+)	16.7693	0.000237648	SPATA6	ssc_circ:chr3:117088766_117089708(-)	ssc_circ:chr3:117088766_117089708(-)	-17.67356	1.22E-08	NA
ssc_circ:chr3:88233932_88295724(+)	ssc_circ:chr3:88233932_88295724(+)	16.6895	0.00043151	NA	ssc_circ:chr9:32266027_32306859(+)	ssc_circ:chr9:32266027_32306859(+)	-17.58439	4.07E-08	ENSSSCG00000047709
circSTRADB	ssc_circ:chr15:105076047_105078519(+)	16.6699	0.00043151	STRADB	circCWH43_2	ssc_circ:chr8:38900358_38928392(+)	-17.39749	5.25E-07	CWH43
circGULP1	ssc_circ:chr15:93239180_93243693(+)	16.6215	0.000761717	GULP1	ssc_circ:chr4:76492549_76546356(-)	ssc_circ:chr4:76492549_76546356(-)	-17.39019	1.71E-06	ENSSSCG00000044763
ssc_circ:chr4:76581881_76640165(-)	ssc_circ:chr4:76581881_76640165(-)	16.6088	0.000761717	ENSSSCG00000046734	circKCTD3_3	ssc_circ:chr10:5777659_5806269(+)	-17.20866	1.91E-05	KCTD3
circTRPM6	ssc_circ:chr1:227918274_227932487(-)	16.604	0.000761717	TRPM6	circLYRM7	ssc_circ:chr2:133634726_133638912(+)	-17.0525	5.76E-06	LYRM7
circLARGE1	ssc_circ:chrAEMK02000361.1:637883_657686(-)	16.5975	0.00138641	LARGE1	circPCCA	ssc_circ:chr11:69070568_69080842(+)	-17.03967	5.76E-06	PCCA
circGFPT1	ssc_circ:chr3:73046115_73059202(+)	16.5927	0.000761717	GFPT1	circDAG1	ssc_circ:chr13:32059508_32059882(+)	-17.03331	1.05E-05	DAG1
circKCTD3_2	ssc_circ:chr10:5761746_5772004(+)	16.5342	0.00138641	KCTD3	ssc_circ:chr5:76899173_76942859(+)	ssc_circ:chr5:76899173_76942859(+)	-17.01197	1.05E-05	ENSSSCG00000050643
circFAM13A	ssc_circ:chr8:130257256_130277496(+)	16.5111	0.00138641	FAM13A	circGRIK1	ssc_circ:chr13:193034630_193049824(-)	-16.97349	1.99E-05	GRIK1
circIGSF23	ssc_circ:chr6:51124303_51145752(-)	16.4961	0.002520468	IGSF23	ssc_circ:chr11:14987360_14987769(-)	ssc_circ:chr11:14987360_14987769(-)	-16.88832	3.81E-05	NA
circEED	ssc_circ:chr9:20136920_20138153(+)	16.4925	0.002520468	EED	circSKA3	ssc_circ:chr11:1317565_1327140(-)	-16.84896	7.06E-05	SKA3
circNUP160	ssc_circ:chr2:14885418_14887371(+)	16.4826	0.002520468	NUP160	circFNBP4	ssc_circ:chr2:14911603_14912714(+)	-16.74224	7.06E-05	FNBP4
circANKRD12	ssc_circ:chr6:98701017_98703045(-)	16.4357	0.004388728	ANKRD12	circFRYL	ssc_circ:chr8:38476237_38478580(-)	-16.71444	0.000237648	FRYL

E: scientific notation.

### GO and KEGG analysis of host genes with differentially expressed circRNAs

**Fig. 5:**
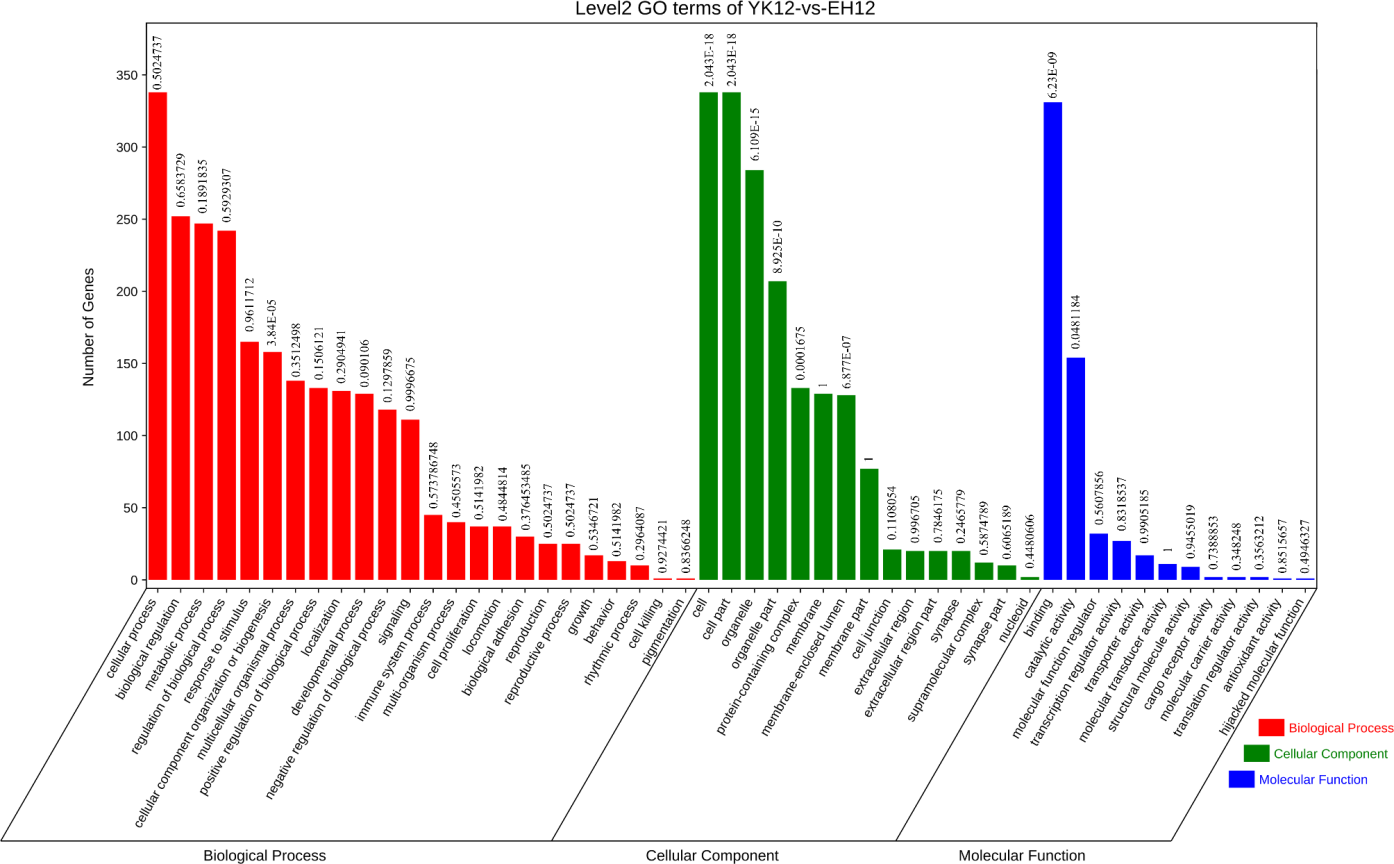
Histogram of GO-enriched classification of DEcircRNAs. The data labels on the bar graph are *q*-values

**Fig. 6:**
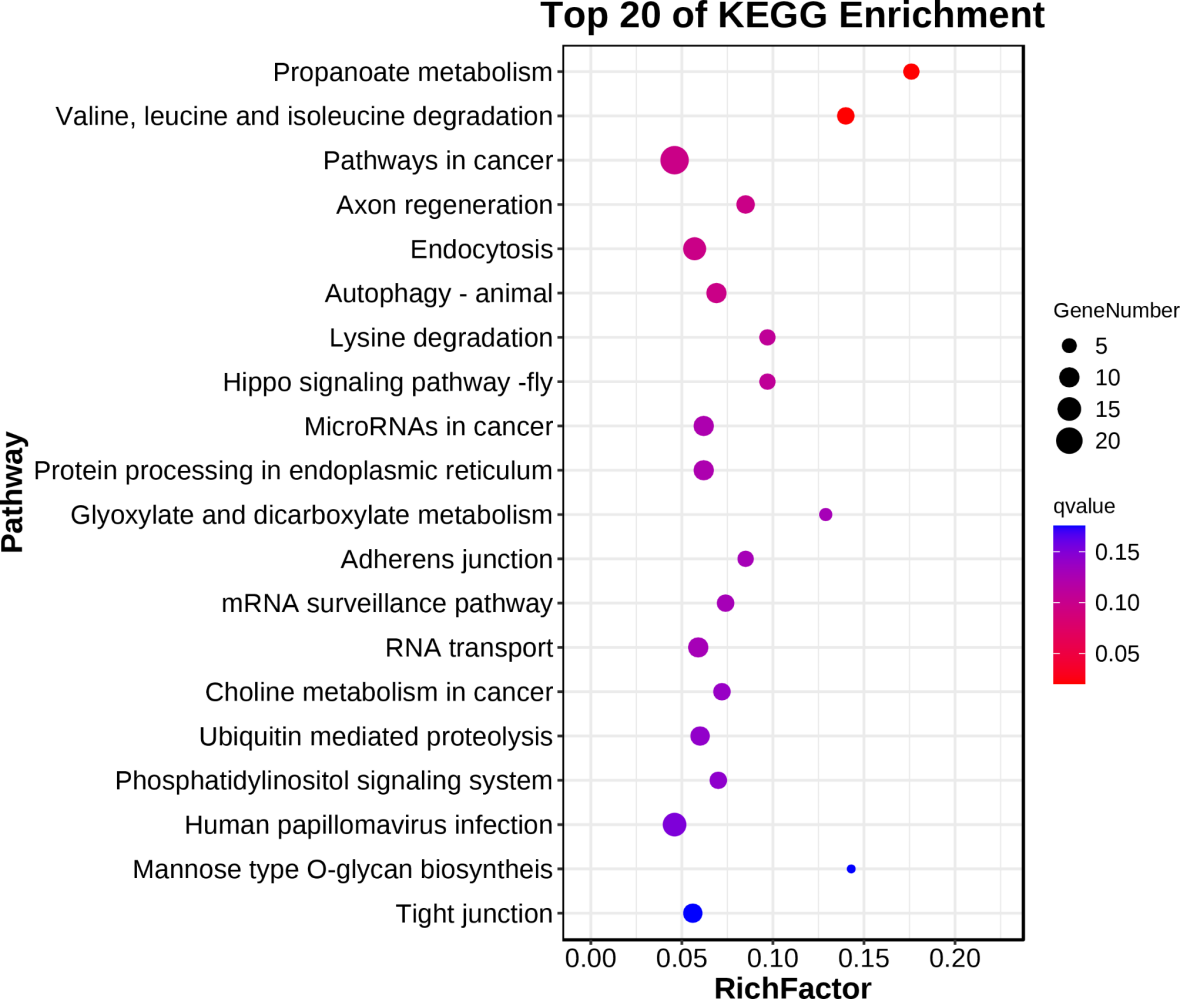
KEGG enrichment scatter plot of DEcircRNAs host genes

Assuming that the functions of circRNAs may be related to the known functions of host genes, we performed GO and KEGG pathway analyses of host genes producing DEcircRNAs to predict their potential functions on the endometrium of Yorkshire and Erhualian pigs at the peri-implantation stage. According to the GO database, the host genes producing DEcircRNAs in Yorkshire pigs and Erhualian pigs were divided into three categories: biological process, cellular component and molecular function (Fig. 5). The host genes of 641 DEcircRNAs generated in the endometrium were significantly enriched to 9 GO entries (*q* < 0.05), including 1 item under “biological process”: “cellular component organization or biogenesis”; 6 items under “cellular component”: “cell”, “cell part”, “organelle”, “organelle part”, “membrane-enclosed lumen”, “protein-containing complex”; 2 items under “molecular function”: “binding”, “catalytic activity” (Supplementary Table S2). Interestingly, many of the genes involved in these pathways were also enriched in adhesion and reproductive processes, indicating their important regulatory roles in implantation. To further understand the role of these DEcircRNAs in the physiological functions of EH12 and YK12 during peri-implantation, we mapped them to terms in the KEGG database (http://www.genome.ad.jp/kegg/). In this study, KEGG pathway annotation revealed that 206 circRNA-hosting genes were annotated into 267 biological processes (Supplementary Table S3). For the pathways of all biological processes, we used the bubble diagram to visualize the number of DEGs related to the top 20 enriched pathways (Fig. 6), of which 2 pathways (“Propanoate metabolism” and “Valine, leucine and isoleucine degradation”) were significantly enriched in the KEGG pathway (*q* < 0.05). These pathways contain 8 genes (ENSSSCG00000001732(MMUT); ENSSSCG00000006866(DBT); ENSSSCG00000009522(PCCA); ENSSSCG00000011653(PCCB); ENSSSCG00000016049 (HIBCH); ENSSSCG00000017198 (ACOX1); ENSSSCG00000009150 (HADH); ENSSSCG00000025942 (MCCC1)), suggesting that these pathways and related genes may play an important role in the regulation of embryo attachment in the peri-implantation endometrium [49, 50].

### Functional prediction of differential circRNAs

**Fig. 7:**
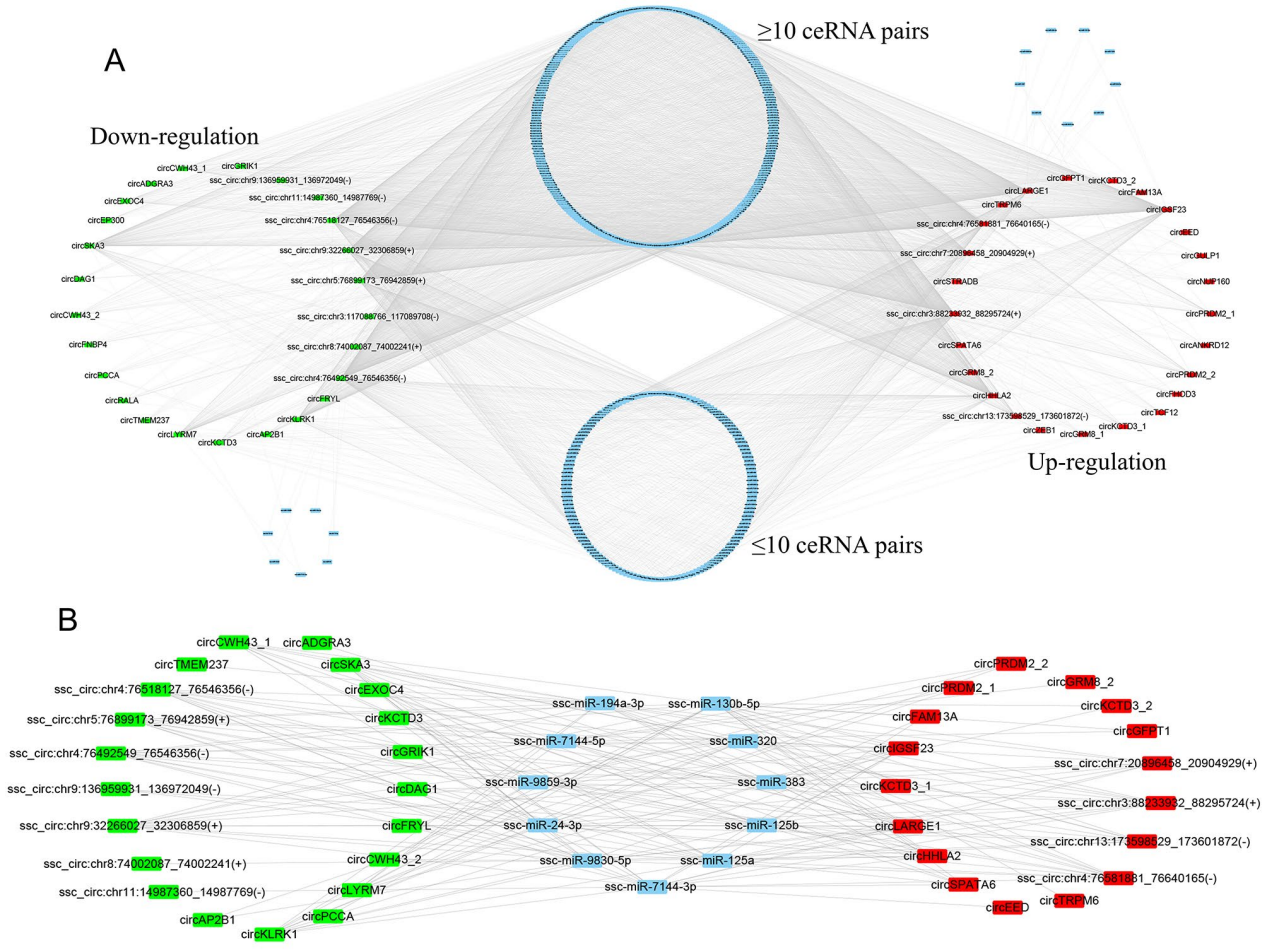
Exploration of circRNA mediated ceRNA regulatory network. (**A**) Illustration of the ceRNA interaction network. Blue nodes represent miRNAs, green nodes represent down-regulated circRNAs (top 25 DEcircRNAs in YK12), and red nodes represent up-regulated circRNAs (top 25 DEcircRNAs in EH12). (**B**) Sub-network of miRNAs with the abundant interactions

It has been previously reported that circRNAs can sponge miRNAs and indirectly regulate gene expression in a post-transcriptional manner [51]. To assess whether all the SDEcircRNAs identified in Erhualian and Yorkshire pig endometrial epithelial cells function as miRNA sponges, we predicted the miRNA targets of these circRNAs using bioinformatics tools. We found that 22,088 (99.91%) of 22,108 circRNAs had miRNA binding sites, while very few circRNAs were predicted to have no potential miRNA targets. Furthermore, we predicted miRNA targets of 641 SDEcircRNAs (Supplementary Table S4). Subsequently, we characterized the interaction network of the top 25 circRNAs significantly differentially and highly expressed in Yorkshire pigs and the top 25 circRNAs significantly differentially highly expressed in Erhualian, and 50 circRNAs formed 4514 interaction relationships with 452 miRNAs (Fig. 7A). Most circRNAs have at least two miRNA binding sites, and each miRNA can also bind to multiple circRNAs, the number of which contains more than 10 circRNA-miRNAs pairs is higher than that of less than 10 circRNA-miRNAs pairs. To further explore the regulation of core circRNA-miRNA pairs, we characterized the first three circRNA-miRNA pairs (21, 18, 17 ceRNA pairs), and obtained 11 miRNAs (ssc-miR-320, ssc-miR-7144-5p, ssc-miR-7144-3p, ssc-miR-9830-5p, ssc-miR-9859-3p, ssc-miR-125b, ssc-miR-24-3p, ssc-miR-125a, ssc-miR-130b- 5p, ssc-miR-383, ssc-miR-194a-3p) (Fig. 7B). In DEcircRNAs, these miRNAs that can bind multiple circRNAs may play important biological roles. Notably, some miRNAs sponged by circRNAs have been shown to regulate endometrial receptivity, embryo attachment and embryonic development. For example, CircRNA-9119 targets miR-26a [52], ciR8073-bound miR181a [53], Circ-8073-bound miR-449a [54], circRNA3175-miR182 [55] in goat endometrial cells, Circ0001470 binds miR-140-3p to promote embryonic development in pigs [56]. Collectively, these results suggest that circRNAs found in porcine endometrial luminal epithelium may have potential miRNA binding sites and play corresponding biological regulatory roles.

**Fig. 8:**
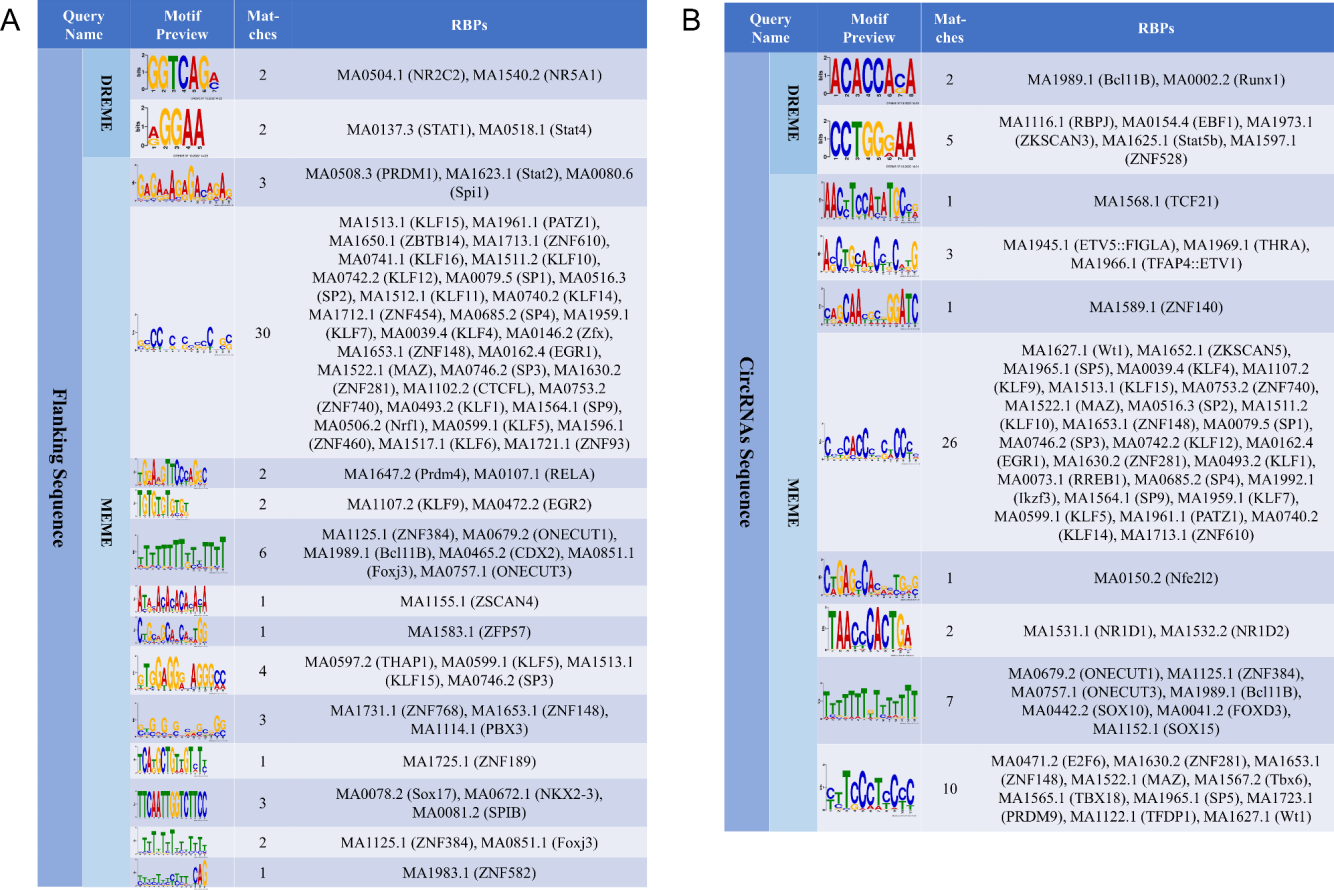
Prediction of differential circRNAs binding proteins. RBP-binding motifs are enriched in flanking regions (**A**) and themselves sequences (**B**) of circRNAs.

To further explore other possible regulatory roles of circRNAs, we performed motif analysis of the flanking sequences and themselves sequences of SDEcircRNAs separately to search for their possible regulatory roles. A total of 63 possible binding proteins were identified by motif analysis of circRNAs flanking sequences (Fig. 8A). A total of 58 possible binding proteins were identified by motif analysis of circRNAs themselves sequences (Fig. 8B). Of these, 25 binding proteins shared flanking sequences with their their self-sequences (Supplementary Table S5). These binding proteins may play important regulatory roles in circRNA production and their function.

### Quantitative validation of differentially expressed circRNAs

**Fig. 9:**
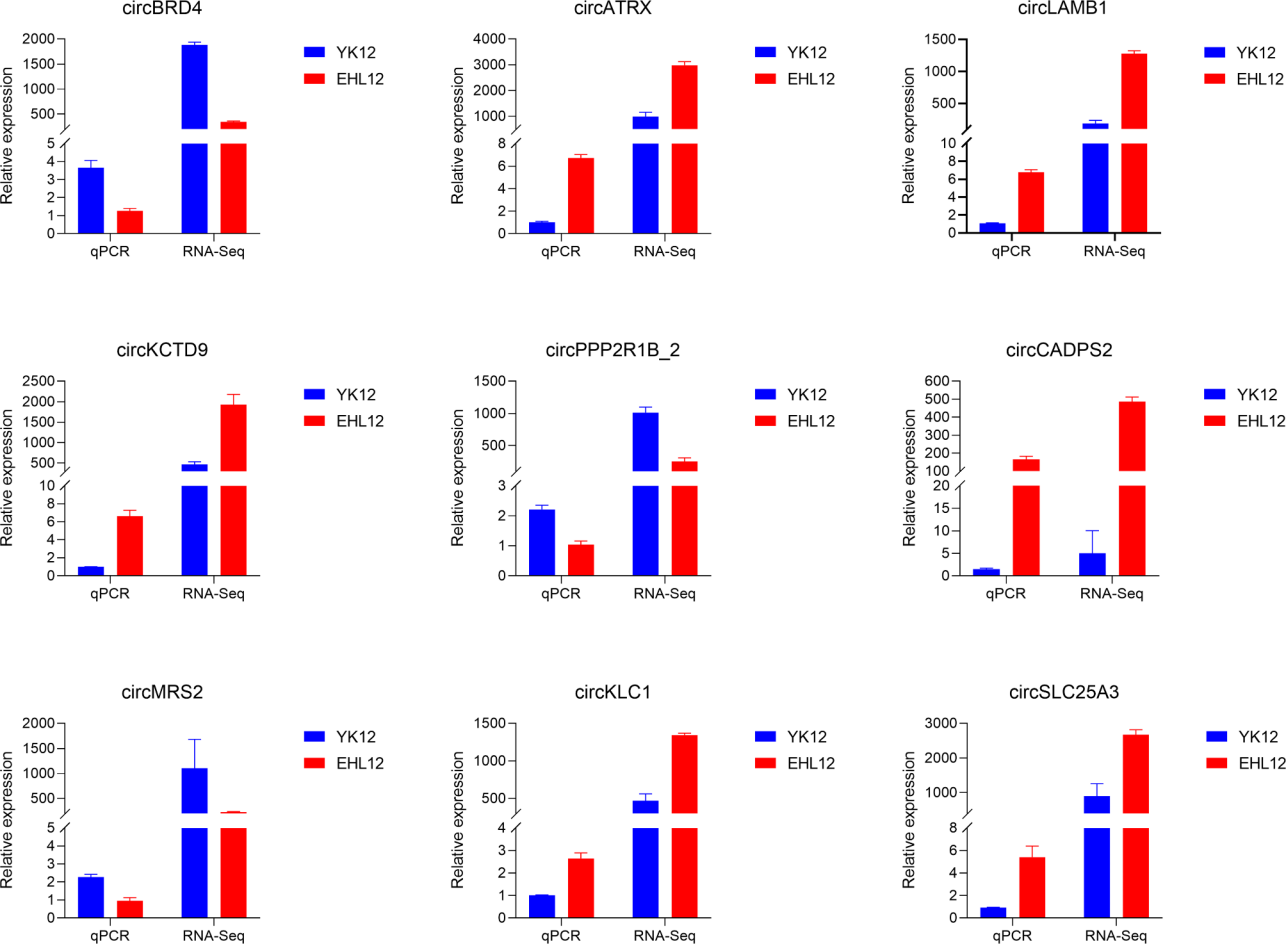
RT-qPCR validation of nine diferentially expressed circRNAs between YK12 and EH12.

In order to verify the reproducibility of RNA-seq data, we randomly selected 9 SDEcircRNAs for RT-qPCR analysis, and these circRNAs were used to verify the presence of circRNAs in YK12 and EH 12 endometrium. As shown in Fig. 9, the expression levels of circATRX, circLAMB1, circKCTD9, circCADPS2, circKLC1 and circSLC25A3 in EH12 endometrium were higher than those in YK12 endometrium. CircBRD4, circPPP2R1B, and circMRS2 gene expression levels were lower in EH12 endometrium compared with YK12 endometrium. The results showed that the RT-qPCR results of these circRNAs in the endometrium were consistent with those obtained by RNA-Seq (Fig. 9), and our RNA-Seq results were reproducible and reliable.

### CircRNAs junction site validation and RNase R resistance validation

**Fig. 10:**
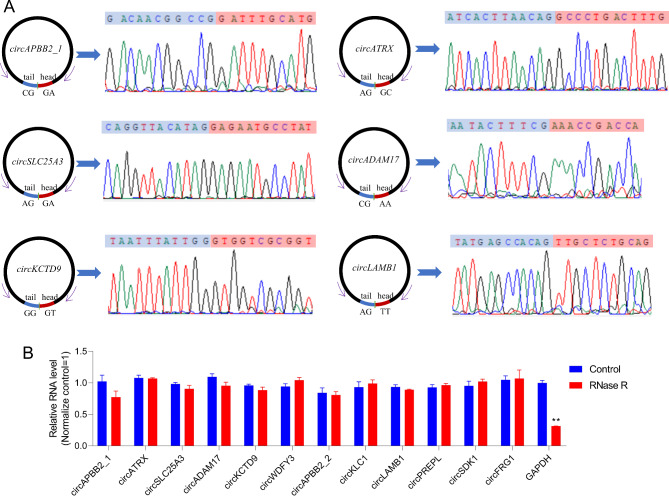
Experimental validation of circRNAs. (**A**) Sanger sequencing to verify the backsplice junction sequence of circRNA. (**B**) Fluorescence quantitative PCR showed that the circRNA was RNase R enzyme resistant. *P < 0.05; **P < 0.01

To verify that the back-splicing events are truly circular, rather than linear trans-splicing products, we examined the physical properties of these products. Six ubiquitously expressed circRNA candidate transcripts were randomly selected to design Divergent primers (Supplementary Table S6). Head-to-tail junction sites were quantified by RT-PCR analysis, and Sanger sequencing further confirmed the presence of head-to-tail splice junctions and the size of these porcine endometrial circRNAs, which was consistent with that provided by RNA-seq (Fig. 10A). In addition, circRNAs were also tested for resistance to RNase R digestion using qPCR (Supplementary Table S6). The expression levels of all the 12 detected circRNAs did not change significantly between the RNase R treatment group and the control group, while the abundance of the linear reference gene GAPDH decreased significantly in the RNaseR treatment group (Fig. 10B), indicating that these circRNAs are all RNase R-resistant.

## Discussion

Litter size is an important economic trait and one of the important factors to improve economic efficiency. In previous studies, we found that Taihu pigs (including Erhualian) had a higher litter size than Yorkshire pigs, which was mainly attributed to the higher embryo implantation rate at the peri-implantation stage [25, 57]. In this study, we compared the expression profiles of circRNAs in the peri-implantation endometrium of EH12 and YK12, and identified for the first time the key factors affecting the differences in the implantation rates of different breeds of pigs. Furthermore, we reconstructed the potential ceRNA regulatory network and predicted binding proteins. Therefore, the analysis of circRNAs in this study can provide a new perspective to study the molecular mechanisms underlying the differences in fertility and spontaneous loss of porcine embryos in different breeds.

CircRNAs can be involved in a variety of biological processes through different mechanisms [58]. Currently, relatively few circRNA studies have been conducted in domestic pigs. In this study, a total of 22,108 circRNAs were identified in 6 endometrial samples, and most of the circRNAs were spliced from exons, mainly distributed on autosomes and X chromosomes. The abundance of circRNAs was significantly different between EH12 and YK12 endometrium, among which 12,168 circRNAs were shared. Our previous study on circRNAs in muscle during porcine embryonic development found that circTUT7 may regulate the generation of circRNAs [59]. Interestingly, in this study, we found that circTUT7 was included in the top 5 highly expressed circRNAs in both EH12 and YK12 endometrium. Based on the same or similar expression patterns of the circRNAs highly expressed in different tissues, we hypothesized that other circRNAs highly expressed in the endometrium of Erhualian and Yorkshire pigs, respectively, may also regulate the biogenesis of circRNAs.

In addition, 641 DEcircRNAs were obtained in EH12 and YK12. In the up-regulation and down-regulation of the top 25 circRNAs, we found that some host genes of circRNAs have important regulatory functions during pregnancy. For example, ENSSSCG00000011025 (ZEB1) [60] is an important regulator of uterine contractions, ENSSSCG00000027935 (FHOD3) [61]and ENSSSCG00000004600 (TCF12) [62] are potential candidates for affecting pig litter size, and ENSSSCG00000014915 (EED) [63] regulates embryo implantation.

To further explore the differences in the regulatory network of embryo implantation between EH12 and YK12, we performed GO and KEGG pathway enrichment analysis of DEcircRNAs host genes. GO enrichment analysis showed significant differences in cell composition, membrane cavity and related protein complexes between the two pig breeds, which may be related to the difference in receptivity of endometrial epithelial cells between Yorkshire and Erhualian during the peri-implantation period [64]. GO enrichment results also showed some cellular catalytic activity and binding terms, which were shown to be associated with porcine embryo implantation [65]. Further, we found that the host genes of these DEcircRNAs are also involved in multiple pregnancy-related biological processes, including " biological adhesion “, “reproductive process” and “growth”. Previous studies have found that MRS2 (ssc_circ:chr7:19265492_19273195(+) host gene) is up-regulated in bovine pregnant endometrium and may be involved in the regulation of maternal recognition of pregnancy [66]. Studies have shown that CDH13(ssc_circ:chr6:5297087_5377909(-) host gene) is up-regulated in the porcine endometrium during implantation and is a gene related to implantation and pregnancy establishment in pigs [67]. PLCB1(ssc_circ:chr17:17026012_17029727(+) host gene) gene plays a role in cell proliferation, cell adhesion, cell growth and survival, and autophagy, and may regulate the change of sheep litter size [68]. ADAMs(ADAM17)(ssc_circ:chr3:126848011_126848971(+) host gene), a disintegrin and metalloproteinase are a family of proteases involved in outer membrane shedding and play a role in various biological processes such as cell adhesion and migration [69, 70]. KEGG analysis involved “Propanoate metabolism” and “Valine, leucine and isoleucine degradation”. Previous studies have shown that Propanoate metabolism is significantly enriched in differentially expressed genes in bovine endometrium during implantation stage [71], and further studies have shown that Propanoate metabolism may play an important role in reproductive hormone regulation [72]. In the “Valine, leucine and isoleucine degradation” pathway, a previous study found that valine increased in the uterine fluid of cows on day 16 of gestation, leucine and isoleucine concentrations decreased on day 16 of the estrous cycle and increased on day 19 of the estrous cycle [73]. Recently, Valine, leucine and isoleucine have been reported to be involved in lipolysis, lipogenesis, glucose metabolism, glucose transport, intestinal barrier function and absorption, milk quality, mammary gland health, early embryonic development and immunity [74].

Many studies have shown that circRNAs can regulate miRNAs by acting as miRNA sponges [36, 45]. Among the top 3 core circRNA-miRNA pairs in this study, we found 11 core miRNAs. Previous studies demonstrated that miR-320 expression was stimulated by steroid hormones and progesterone during the implantation window to regulate embryo implantation in mice [75]. In humans, miR-24-3p obtained from endometrial fluid can be used as one of the markers for the successful recognition of embryo implantation in the endometrium [76]. The expression level of miR-125a was significantly decreased in maternal plasma of patients with RSA [77–79]. However, overexpression of some of these miRNAs may lead to pregnancy failure. For example, it was shown that placenta-specific miR-125b overexpression resulted in increased pregnancy loss in mice [80]. Studies have shown that miR-383 is upregulated in RPL patients and inhibits LIG4 expression by interacting with the 3’UTR of LIG4 mRNA, and that downregulation of LIG1 and LIG4 increases DNA damage in trophoblast cells, which further induces apoptosis and cell cycle arrest [81]. Further high-throughput characterization of the miRNA and mRNA landscape between YK12 and EH12 endometrium is needed in subsequent studies to explore their potential regulatory axes.

CircRNAs can interact with proteins to perform their biological functions [82]. RBPs are involved in cellular processes such as proliferation, differentiation, translocation, apoptosis, senescence, and post-transcriptional oxidative regulation [83]. In this study, circRNAs sequence and flanking sequence were used to predict the binding proteins. Some of these binding proteins were previously found to be important in regulating embryo development and implantation during pregnancy. For example, the transcription factor EGR1 was found to be estrogen-induced and essential for embryo implantation in the mouse uterus [84, 85]. Previous research data support that Sp1 is involved in the expression of endometrial epithelial-specific genes and may play an important regulatory role in early pregnancy [86]. In humans, MAZ is a transcription factor required for trophoblast self-renewal [87]. Zfp281 is a conserved factor that maintains the plasticity of mouse trophoblast stem cells [88]. PATZ1 can regulate mouse cell growth and embryonic development [89]. Onecut 1 is a potential regulator of mouse embryonic retinal development [90, 91]. Interestingly, among the differential circRNAs binding proteins in this study, we were surprised to find that the Kruppel-like transcription factor (KLF) family genes play an important regulatory role in embryonic development and implantation. KLF1 was found to positively regulate mouse embryonic β-globin expression and has additional overlapping roles in embryonic primitive erythropoiesis [92]. The transcription factor Klf4 can regulate the differentiation of mouse perinatal fibroblasts and myofibroblasts [93]. Klf5 was found to be a marker gene for the expression of embryonic trophoblast stem cells from isolated mouse trophoblast stem cells [94]. In bovine endometrium, KLF5 participates in endometrial remodeling by regulating angiogenesis [95]. In a genome-wide association study in sheep, KLF5 was found to affect embryonic development and litter size [96]. KLF7 is an important transcription factor regulating the development of mouse embryonic olfactory bulb [97]. The transcription factor Klf9 was found to be a progesterone receptor (PGR) coactivator in the uterus, and Klf9-deficient mice exhibited low fertility and reduced progesterone sensitivity [98]. While klf12 plays a negative role in pregnancy, klf12 plays a negative role in pregnancy, and studies have found that klf12 overexpression can cause fetal death and neural tube development disorders in mice [99]. Further studies revealed that KLF12 disrupted endometrial decidualization through transcriptional repression of Nur77 [100]. Klf14 is an imprinted transcription factor that regulates mouse placental growth [101]. Klf15 expression can inhibit the proliferation of uterine epithelial cells in the peri-implantation mice [102], which may be beneficial to the formation of endometrial receptivity. In addition, KLF10 and some members of the zinc finger protein (ZNF) family are also binding factors of circRNAs sequence and flanking sequence, and their physiological regulatory mechanisms in the growth and development of mammalian embryos need to be further explored. Therefore, this study demonstrates that DEcircRNAs and their binding proteins play important regulatory roles during the peri-implantation period.

## Conclusions

In summary, we obtained high-quality circRNA expression profiles from the endometrial tissues of peri-implantation Erhualian and Yorkshire pigs. We identified 22,108 circRNAs, 641 of which were differentially expressed between EH12 and YK12. GO and KEGG analysis, circRNA-miRNA interaction network analysis, and circRNA-binding protein analysis contribute to a better understanding of how circRNAs mediate the regulation of target genes during the peri-implantation phase. The results described in this study may provide reference material for animal reproductive regulation and animal breeding.

## Electronic supplementary material

Below is the link to the electronic supplementary material.

Supplementary Material 1

Supplementary Material 2

Supplementary Material 3

Supplementary Material 4

Supplementary Material 5

Supplementary Material 6

## Data Availability

Raw RNA-Seq data of Erhualian and Yorkshire pig endometrial circRNAs are available in the NCBI database with Bioproject accession number PRJNA945199 (https://www.ncbi.nlm.nih.gov/bioproject/PRJNA945199). All data generated or analyzed in this study are included in this paper and its Supplementary Information files.

## Abbreviations

EH: Erhualian
EH12: Erhualian Pigs on Day 12 of Gestation
YK: Yorkshire
YK12: Yorkshire Pigs on Day 12 of Gestation
CircRNA: Circular RNA
SDE: Significant Differentially Expressed
TL: Taihu Lake
H&E: Hematoxylin-Eosin Staining
Mers: Monomer Units
GO: Gene Ontology
FDR: False Discovery Rate
KEGG: Kyoto Encyclopedia of Genes and Genomes
RT-PCR: Reverse Transcription PCR
qPCR: Quantitative Real-Time PCR
PCA: Principal Component Analysis

## References

[1] Wähner M, Brüssow K (2009). Biological potential of fecundity of sows. Biotechnol Anim Husb.

[2] Argente MJ. Chap. 4 Major Components in Limiting Litter Size. In: *2018*; 2018.

[3] Malopolska MM, Tuz R, Schwarz T, Ekanayake LD, D’Ambrosio J, Ahmadi B, Nowicki J, Tomaszewska E, Grzesiak M, Bartlewski PM (2021). Correlates of reproductive tract anatomy and uterine histomorphometrics with fertility in swine. Theriogenology.

[4] Ye TM, Pang RT, Leung CO, Chiu JF, Yeung WS (2015). Two-dimensional liquid chromatography with tandem mass spectrometry-based proteomic characterization of endometrial luminal epithelial surface proteins responsible for embryo implantation. Fertil Steril.

[5] Geisert RD, Johnson GA, Burghardt RC. Implantation and establishment of pregnancy in the Pig. *Advances in anatomy, embryology, and cell biology* 2015, 216:137–63. 10.1007/978-3-319-15856-3_8 26450498

[6] Wilson ME, Ford SP (1997). Differences in trophectoderm mitotic rate and P450 17alpha-hydroxylase expression between late preimplantation Meishan and Yorkshire conceptuses. Biol Reprod.

[7] Waclawik A, Kaczmarek MM, Blitek A, Kaczynski P, Ziecik AJ (2017). Embryo-maternal dialogue during pregnancy establishment and implantation in the pig. Mol Reprod Dev.

[8] Young KH, Kraeling RR, Bazer FW (1990). Effect of pregnancy and exogenous ovarian steroids on endometrial prolactin receptor ontogeny and uterine secretory response in pigs. Biol Reprod.

[9] Igwebuike UM (2009). A review of uterine structural modifications that influence conceptus implantation and development in sheep and goats. Anim Reprod Sci.

[10] Burghardt RC, Bowen JA, Newton GR, Bazer FW (1997). Extracellular matrix and the implantation cascade in pigs. J Reprod fertility Supplement.

[11] Thie M, Denker HW (2002). In vitro studies on endometrial adhesiveness for trophoblast: cellular dynamics in uterine epithelial cells. Cells Tissues Organs.

[12] Lessey BA, Young SL (2019). What exactly is endometrial receptivity?. Fertil Steril.

[13] Ren Q, Guan S, Fu J, Wang A (2011). Expression of tissue inhibitor of metalloproteinases-3 messenger RNA and protein in porcine endometrium during implantation. Mol Biol Rep.

[14] Ren Q, Guan S, Fu J, Wang A (2010). Temporal and spatial expression of Muc1 during implantation in sows. Int J Mol Sci.

[15] Li W, Xi Y, Xue S, Wang Y, Wu L, Liu H, Lei M (2018). Sequence analysis of microRNAs during pre-implantation between Meishan and Yorkshire pigs. Gene.

[16] Yelich JV, Pomp D, Geisert RD (1997). Detection of transcripts for retinoic acid receptors, retinol-binding protein, and transforming growth factors during rapid trophoblastic elongation in the porcine conceptus. Biol Reprod.

[17] Spencer TE (2013). Early pregnancy: concepts, challenges, and potential solutions. Anim Front.

[18] Huang T, Zhang M, Yan G, Huang X, Chen H, Zhou L, Deng W, Zhang Z, Qiu H, Ai H (2019). Genome-wide association and evolutionary analyses reveal the formation of swine facial wrinkles in chinese erhualian pigs. Aging.

[19] Liu C, Li P, Zhou W, Ma X, Wang X, Xu Y, Jiang N, Zhao M, Zhou T, Yin Y (2020). Genome Data Uncover Conservation Status, historical relatedness and candidate genes under selection in Chinese Indigenous Pigs in the Taihu Lake Region. Front Genet.

[20] Haley CS, Lee GJ (1993). Genetic basis of prolificacy in Meishan pigs. J Reprod fertility Supplement.

[21] Galvin JM, Wilmut I, Day BN, Ritchie M, Thomson M, Haley CS (1993). Reproductive performance in relation to uterine and embryonic traits during early gestation in Meishan, large white and crossbred sows. J Reprod Infertil.

[22] Ashworth CJ, Pickard AR, Miller SJ, Flint AP, Diehl JR (1997). Comparative studies of conceptus-endometrial interactions in large white x landrace and Meishan gilts. Reprod Fertil Dev.

[23] Christenson RK, Vallet JL, Leymaster KA, Young LD (1993). Uterine function in Meishan pigs. J Reprod fertility Supplement.

[24] Rothschild M, Jacobson C, Vaske D, Tuggle C, Wang L, Short T, Eckardt G, Sasaki S, Vincent A, McLaren D (1996). The estrogen receptor locus is associated with a major gene influencing litter size in pigs. Proc Natl Acad Sci USA.

[25] Gu T, Zhu MJ, Schroyen M, Qu L, Nettleton D, Kuhar D, Lunney JK, Ross JW, Zhao SH, Tuggle CK (2014). Endometrial gene expression profiling in pregnant Meishan and Yorkshire pigs on day 12 of gestation. BMC Genomics.

[26] Wessels JM, Linton NF, Croy BA, Tayade C (2007). A review of molecular contrasts between arresting and viable porcine attachment sites. Am J Reprod Immunol.

[27] Fu Y, Fu J, Wang A (2012). Association of EphA4 polymorphism with swine reproductive traits and mRNA expression of EphA4 during embryo implantation. Mol Biol Rep.

[28] Wu YP, Wang AG, Li N, Fu JL, Zhao XB (2010). Association with TGF-beta1 gene polymorphisms and reproductive performance of large white pig. Reprod Domest animals = Zuchthygiene.

[29] Chen X, Li A, Chen W, Wei J, Fu J, Wang A (2015). Differential gene expression in uterine endometrium during implantation in pigs. Biol Reprod.

[30] Zhang C, Hu J, Yu Y (2020). CircRNA is a rising star in Researches of Ocular Diseases. Front cell Dev biology.

[31] Patop IL, Wust S, Kadener S (2019). Past, present, and future of circRNAs. EMBO J.

[32] Verduci L, Tarcitano E, Strano S, Yarden Y, Blandino G (2021). CircRNAs: role in human diseases and potential use as biomarkers. Cell Death Dis.

[33] Liu KS, Pan F, Mao XD, Liu C, Chen YJ (2019). Biological functions of circular RNAs and their roles in occurrence of reproduction and gynecological diseases. Am J translational Res.

[34] Min X, Liu DL, Xiong XD (2021). Circular RNAs as competing endogenous RNAs in Cardiovascular and Cerebrovascular Diseases: Molecular Mechanisms and clinical implications. Front Cardiovasc Med.

[35] Das A, Sinha T, Shyamal S, Panda AC. Emerging role of circular RNA-Protein interactions. Non-coding RNA 2021, 7(3). 10.3390/ncrna7030048 PMC8395946 34449657

[36] Zheng Q, Bao C, Guo W, Li S, Chen J, Chen B, Luo Y, Lyu D, Li Y, Shi G (2016). Circular RNA profiling reveals an abundant circHIPK3 that regulates cell growth by sponging multiple miRNAs. Nat Commun.

[37] Song M, Zhao G, Sun H, Yao S, Zhou Z, Jiang P, Wu Q, Zhu H, Wang H, Dai C et al. circPTPN12/miR-21-5 p/∆Np63alpha pathway contributes to human endometrial fibrosis. eLife 2021, 10. 10.7554/eLife.65735 PMC8208816 34132637

[38] Li Z, Shi L, Li Q, Zhao J, Lu W, Wang J. The expression and Bioinformatics Analysis of Circular RNAs in Endometritis Mouse Uterus Tissues. Molecules 2022, 27(12). 10.3390/molecules27123682 PMC9230989 35744807

[39] Song Y, Zhang L, Liu X, Niu M, Cui J, Che S, Liu Y, An X, Cao B (2019). Analyses of circRNA profiling during the development from pre-receptive to receptive phases in the goat endometrium. J Anim Sci Biotechnol.

[40] Huang Y, Zhang C, Xiong J, Ren H (2021). Emerging important roles of circRNAs in human cancer and other diseases. Genes & diseases.

[41] Tu J, Yang H, Chen Y, Chen Y, Chen H, Li Z, Li L, Zhang Y, Chen X, Yu Z (2021). Current and future roles of circular RNAs in normal and pathological endometrium. Front Endocrinol.

[42] Chen S, Zhou Y, Chen Y, Gu J (2018). Fastp: an ultra-fast all-in-one FASTQ preprocessor. Bioinformatics.

[43] Langmead B, Salzberg SL (2012). Fast gapped-read alignment with Bowtie 2. Nat Methods.

[44] Kim D, Pertea G, Trapnell C, Pimentel H, Kelley R, Salzberg SL (2013). TopHat2: accurate alignment of transcriptomes in the presence of insertions, deletions and gene fusions. Genome Biol.

[45] Memczak S, Jens M, Elefsinioti A, Torti F, Krueger J, Rybak A, Maier L, Mackowiak SD, Gregersen LH, Munschauer M (2013). Circular RNAs are a large class of animal RNAs with regulatory potency. Nature.

[46] Zhang XO, Wang HB, Zhang Y, Lu X, Chen LL, Yang L (2014). Complementary sequence-mediated exon circularization. Cell.

[47] The Gene Ontology C (2017). Expansion of the Gene Ontology knowledgebase and resources. Nucleic Acids Res.

[48] Kanehisa M, Araki M, Goto S, Hattori M, Hirakawa M, Itoh M, Katayama T, Kawashima S, Okuda S, Tokimatsu T (2008). KEGG for linking genomes to life and the environment. Nucleic Acids Res.

[49] Ji Y, Lu X, Zhong Q, Liu P, An Y, Zhang Y, Zhang S, Jia R, Tesfamariam IG, Kahsay AG (2013). Transcriptional profiling of mouse uterus at pre-implantation stage under VEGF repression. PLoS ONE.

[50] Aikawa S, Hirota Y, Fukui Y, Ishizawa C, Kaku RII, Hirata T, Akaeda T, Hiraoka S, Matsuo T (2022). A gene network of uterine luminal epithelium organizes mouse blastocyst implantation. Reproductive Med biology.

[51] Tang X, Ren H, Guo M, Qian J, Yang Y, Gu C (2021). Review on circular RNAs and new insights into their roles in cancer. Comput Struct Biotechnol J.

[52] Zhang L, Liu X, Che S, Cui J, Liu Y, An X, Cao B, Song Y (2018). CircRNA-9119 regulates the expression of prostaglandin-endoperoxide synthase 2 (PTGS2) by sponging miR-26a in the endometrial epithelial cells of dairy goat. Reprod Fertil Dev.

[53] Zhang L, Liu X, Che S, Cui J, Ma X, An X, Cao B, Song Y (2019). Endometrial epithelial cell apoptosis is inhibited by a ciR8073-miR181a-Neurotensis pathway during embryo implantation. Mol therapy Nucleic acids.

[54] Liu X, Zhang L, Liu Y, Cui J, Che S, An X, Song Y, Cao B (2018). Circ-8073 regulates CEP55 by sponging miR-449a to promote caprine endometrial epithelial cells proliferation via the PI3K/AKT/mTOR pathway. Biochim et Biophys acta Mol cell Res.

[55] Zhang L, Liu X, Ma X, Liu Y, Che S, Cui J, An X, Cao B, Song Y (2018). Testin was regulated by circRNA3175-miR182 and inhibited endometrial epithelial cell apoptosis in pre-receptive endometrium of dairy goats. J Cell Physiol.

[56] Zhang L, Zhou C, Jiang X, Huang S, Li Y, Su T, Wang G, Zhou Y, Liu M, Xu D. Circ0001470 Acts as a mir-140-3p sponge to facilitate the progression of Embryonic Development through regulating PTGFR expression. Cells 2022, 11(11). 10.3390/cells11111746 PMC9179393 35681442

[57] Zhang H, Wang S, Liu M, Zhang A, Wu Z, Zhang Z, Li J (2013). Differential gene expression in the endometrium on gestation day 12 provides insight into sow prolificacy. BMC Genomics.

[58] Yu CY, Kuo HC (2019). The emerging roles and functions of circular RNAs and their generation. J Biomed Sci.

[59] Hong L, Gu T, He Y, Zhou C, Hu Q, Wang X, Zheng E, Huang S, Xu Z, Yang J (2019). Genome-wide analysis of circular RNAs mediated ceRNA regulation in porcine embryonic muscle development. Front cell Dev biology.

[60] Renthal NE, Chen CC, Williams KC, Gerard RD, Prange-Kiel J, Mendelson CR (2010). miR-200 family and targets, ZEB1 and ZEB2, modulate uterine quiescence and contractility during pregnancy and labor. Proc Natl Acad Sci USA.

[61] Ding R, Qiu Y, Zhuang Z, Ruan D, Wu J, Zhou S, Ye J, Cao L, Hong L, Xu Z (2021). Genome-wide association studies reveals polygenic genetic architecture of litter traits in Duroc pigs. Theriogenology.

[62] Tao H, Mei S, Sun X, Peng X, Zhang X, Ma C, Wang L, Hua L, Li F (2013). Associations of TCF12, CTNNAL1 and WNT10B gene polymorphisms with litter size in pigs. Anim Reprod Sci.

[63] Saha B, Home P, Ray S, Larson M, Paul A, Rajendran G, Behr B, Paul S (2013). EED and KDM6B coordinate the first mammalian cell lineage commitment to ensure embryo implantation. Mol Cell Biol.

[64] Hua R, Wang Y, Lian W, Li W, Xi Y, Xue S, Kang T, Lei M (2021). Small RNA-seq analysis of extracellular vesicles from porcine uterine flushing fluids during peri-implantation. Gene.

[65] Wang Y, Xue S, Liu X, Liu H, Hu T, Qiu X, Zhang J, Lei M (2016). Analyses of long non-coding RNA and mRNA profiling using RNA sequencing during the pre-implantation phases in pig endometrium. Sci Rep.

[66] Adhikari B, Lee CN, Khadka VS, Deng Y, Fukumoto G, Thorne M, Caires K, Odani J, Mishra B (2022). RNA-Sequencing based analysis of bovine endometrium during the maternal recognition of pregnancy. BMC Genomics.

[67] Goryszewska E, Kaczynski P, Baryla M, Waclawik A (2021). Pleiotropic role of prokineticin 1 in the porcine endometrium during pregnancy establishment and embryo implantation dagger. Biol Reprod.

[68] Gholizadeh M, Esmaeili-Fard SM (2022). Meta-analysis of genome-wide association studies for litter size in sheep. Theriogenology.

[69] Kawaguchi M, Hozumi Y, Suzuki T (2015). ADAM protease inhibitors reduce melanogenesis by regulating PMEL17 processing in human melanocytes. J Dermatol Sci.

[70] Takahashi H, Yuge K, Matsubara S, Ohkuchi A, Kuwata T, Usui R, Suzuki M, Takizawa T (2014). Differential expression of ADAM (a disintegrin and metalloproteinase) genes between human first trimester villous and extravillous trophoblast cells. J Nippon Med School = Nippon Ika Daigaku zasshi.

[71] Mansouri-Attia N, Aubert J, Reinaud P, Giraud-Delville C, Taghouti G, Galio L, Everts RE, Degrelle S, Richard C, Hue I (2009). Gene expression profiles of bovine caruncular and intercaruncular endometrium at implantation. Physiol Genom.

[72] Bedford A, Beckett L, Hardin K, Dias NW, Davis T, Mercadante VRG, Ealy AD, White RR (2018). Propionate affects insulin signaling and progesterone profiles in dairy heifers. Sci Rep.

[73] Forde N, Simintiras CA, Sturmey R, Mamo S, Kelly AK, Spencer TE, Bazer FW, Lonergan P (2014). Amino acids in the uterine luminal fluid reflects the temporal changes in transporter expression in the endometrium and conceptus during early pregnancy in cattle. PLoS ONE.

[74] Zhang S, Zeng X, Ren M, Mao X, Qiao S (2017). Novel metabolic and physiological functions of branched chain amino acids: a review. J Anim Sci Biotechnol.

[75] Xia HF, Jin XH, Song PP, Cui Y, Liu CM, Ma X (2010). Temporal and spatial regulation of miR-320 in the uterus during embryo implantation in the rat. Int J Mol Sci.

[76] Ibanez-Perez J, Diaz-Nunez M, Clos-Garcia M, Lainz L, Iglesias M, Diez-Zapirain M, Rabanal A, Barcena L, Gonzalez M, Lozano JJ (2022). microRNA-based signatures obtained from endometrial fluid identify implantative endometrium. Hum Reprod.

[77] Hu Y, Liu CM, Qi L, He TZ, Shi-Guo L, Hao CJ, Cui Y, Zhang N, Xia HF, Ma X (2011). Two common SNPs in pri-miR-125a alter the mature miRNA expression and associate with recurrent pregnancy loss in a Han-Chinese population. RNA Biol.

[78] Hu Y, Huo ZH, Liu CM, Liu SG, Zhang N, Yin KL, Qi L, Ma X, Xia HF (2014). Functional study of one nucleotide mutation in pri-miR-125a coding region which related to recurrent pregnancy loss. PLoS ONE.

[79] Manzoor U, Pandith AA, Amin I, Wani S, Sanadhya D, Lone TA, Mir H, Paray BA, Gulnaz A, Anwar I et al. Implications of decreased expression of miR-125a with respect to its variant allele in the pathogenesis of recurrent pregnancy loss: a study in a high incidence zone. J Clin Med 2022, 11(13). 10.3390/jcm11133834 PMC9267497 35807118

[80] Sun F, Cai H, Tan L, Qin D, Zhang J, Hua J, Fan X, Peng S. Placenta-specific miR-125b overexpression leads to increased rates of pregnancy loss in mice. Int J Mol Sci 2022, 23(2). 10.3390/ijms23020943 PMC8779150 35055127

[81] Li PF, Xiang YG, Zhang D, Lu N, Dou Q, Tan L (2021). Downregulation of DNA ligases in trophoblasts contributes to recurrent pregnancy loss through inducing DNA damages. Placenta.

[82] Zhou WY, Cai ZR, Liu J, Wang DS, Ju HQ, Xu RH (2020). Circular RNA: metabolism, functions and interactions with proteins. Mol Cancer.

[83] Abdelmohsen K, Kuwano Y, Kim HH, Gorospe M (2008). Posttranscriptional gene regulation by RNA-binding proteins during oxidative stress: implications for cellular senescence. Biol Chem.

[84] Park M, Park SH, Park H, Kim HR, Lim HJ, Song H (2021). ADAMTS-1: a novel target gene of an estrogen-induced transcription factor, EGR1, critical for embryo implantation in the mouse uterus. Cell & bioscience.

[85] Kim HR, Kim YS, Yoon JA, Yang SC, Park M, Seol DW, Lyu SW, Jun JH, Lim HJ, Lee DR (2018). Estrogen induces EGR1 to fine-tune its actions on uterine epithelium by controlling PR signaling for successful embryo implantation. FASEB journal: official publication of the Federation of American Societies for Experimental Biology.

[86] Simmen RC, Zhang XL, Zhang D, Wang Y, Michel FJ, Simmen FA (2000). Expression and regulatory function of the transcription factor Sp1 in the uterine endometrium at early pregnancy: implications for epithelial phenotype. Mol Cell Endocrinol.

[87] Chen Y, Siriwardena D, Penfold C, Pavlinek A, Boroviak TE. An integrated atlas of human placental development delineates essential regulators of trophoblast stem cells. Development 2022, 149(13). 10.1242/dev.200171 PMC9340556 35792865

[88] Ishiuchi T, Ohishi H, Sato T, Kamimura S, Yorino M, Abe S, Suzuki A, Wakayama T, Suyama M, Sasaki H (2019). Zfp281 shapes the transcriptome of trophoblast stem cells and is essential for placental development. Cell Rep.

[89] Valentino T, Palmieri D, Vitiello M, Simeone A, Palma G, Arra C, Chieffi P, Chiariotti L, Fusco A, Fedele M (2013). Embryonic defects and growth alteration in mice with homozygous disruption of the Patz1 gene. J Cell Physiol.

[90] Wu F, Sapkota D, Li R, Mu X (2012). Onecut 1 and onecut 2 are potential regulators of mouse retinal development. J Comp Neurol.

[91] Sapkota D, Chintala H, Wu F, Fliesler SJ, Hu Z, Mu X (2014). Onecut1 and Onecut2 redundantly regulate early retinal cell fates during development. Proc Natl Acad Sci USA.

[92] Pang CJ, Lemsaddek W, Alhashem YN, Bondzi C, Redmond LC, Ah-Son N, Dumur CI, Archer KJ, Haar JL, Lloyd JA (2012). Kruppel-like factor 1 (KLF1), KLF2, and myc control a regulatory network essential for embryonic erythropoiesis. Mol Cell Biol.

[93] Jean JC, George E, Kaestner KH, Brown LA, Spira A, Joyce-Brady M (2013). Transcription factor Klf4, induced in the lung by oxygen at birth, regulates perinatal fibroblast and myofibroblast differentiation. PLoS ONE.

[94] Ohinata Y, Tsukiyama T (2014). Establishment of trophoblast stem cells under defined culture conditions in mice. PLoS ONE.

[95] Mitko K, Ulbrich SE, Wenigerkind H, Sinowatz F, Blum H, Wolf E, Bauersachs S (2008). Dynamic changes in messenger RNA profiles of bovine endometrium during the oestrous cycle. Reproduction.

[96] Tao L, He XY, Wang FY, Pan LX, Wang XY, Gan SQ, Di R, Chu MX (2021). Identification of genes associated with litter size combining genomic approaches in Luzhong mutton sheep. Anim Genet.

[97] Laub F, Dragomir C, Ramirez F (2006). Mice without transcription factor KLF7 provide new insight into olfactory bulb development. Brain Res.

[98] Heard ME, Pabona JM, Clayberger C, Krensky AM, Simmen FA, Simmen RC (2012). The reproductive phenotype of mice null for transcription factor kruppel-like factor 13 suggests compensatory function of family member kruppel-like factor 9 in the peri-implantation uterus. Biol Reprod.

[99] Liu Y, Yuan Q, Wang Z, Ding L, Kong N, Liu J, Hu Y, Zhang Y, Li C, Yan G (2021). A high level of KLF12 causes folic acid-resistant neural tube defects by activating the shh signaling pathway in micedagger. Biol Reprod.

[100] Huang C, Jiang Y, Zhou J, Yan Q, Jiang R, Cheng X, Xing J, Ding L, Sun J, Yan G (2017). Increased kruppel-like factor 12 in recurrent implantation failure impairs endometrial decidualization by repressing Nur77 expression. Reproductive biology and endocrinology: RB&E.

[101] Koppes E, Shaffer B, Sadovsky E, Himes K, Barak Y, Sadovsky Y, Chaillet JR (2019). Klf14 is an imprinted transcription factor that regulates placental growth. Placenta.

[102] Kim TH, Yoo JY, Wang Z, Lydon JP, Khatri S, Hawkins SM, Leach RE, Fazleabas AT, Young SL, Lessey BA (2015). ARID1A is essential for endometrial function during early pregnancy. PLoS Genet.

